# Cancer survivorship programs for patients from culturally and linguistically diverse (CALD) backgrounds: a scoping review

**DOI:** 10.1007/s11764-023-01442-w

**Published:** 2023-08-12

**Authors:** Lawrence Kasherman, Won-Hee Yoon, Sim Yee (Cindy) Tan, Ashanya Malalasekera, Joanne Shaw, Janette Vardy

**Affiliations:** 1 Concord Clinical School, Faculty of Medicine and Health, University of Sydney, Sydney, NSW 2138 Australia; 2 Department of Medical Oncology, Illawarra Cancer Care Centre, Wollongong, NSW Australia; 3 Sydney Cancer Survivorship Centre, Department of Medical Oncology, Concord Hospital, Concord, NSW Australia; 4 Westmead Clinical School, Faculty of Medicine and Health, University of Sydney, Sydney, NSW Australia; 5 Psycho-Oncology Co-operative Research Group (PoCoG), School of Psychology, University of Sydney, Sydney, NSW Australia

**Keywords:** Culturally and linguistically diverse, Survivorship, Scoping review

## Abstract

**Purpose:**

People of Culturally and Linguistically Diverse (CALD) backgrounds face disparities in cancer care. This scoping review aims to identify the breadth of international literature focused on cancer survivorship programs/interventions specific to CALD populations, and barriers and facilitators to program participation.

**Methods:**

Scoping review included studies focused on interventions for CALD cancer survivors after curative-intent treatment. Electronic databases: Medline, Embase, CINAHL, PsycInfo and Scopus were searched, for original research articles from database inception to April 2022.

**Results:**

710 references were screened with 26 included: 14 randomized (54%), 6 mixed-method (23%), 4 non-randomized experimental (15%), 2 qualitative studies (8%). Most were United States-based (85%), in breast cancer survivors (88%; Table 1), of Hispanic/Latinx (54%) and Chinese (27%) backgrounds. Patient-reported outcome measures were frequently incorporated as primary endpoints (65%), or secondary endpoints (15%). 81% used multi-modal interventions with most encompassing domains of managing psychosocial (85%) or physical (77%) effects from cancer, and most were developed through community-based participatory methods (46%) or informed by earlier work by the same research groups (35%). Interventions were usually delivered by bilingual staff (88%). 17 studies (77%) met their primary endpoints, such as meeting feasibility targets or improvements in quality of life or psychological outcomes. Barriers and facilitators included cultural sensitivity, health literacy, socioeconomic status, acculturation, and access.

**Conclusions:**

Positive outcomes were associated with cancer survivorship programs/interventions for CALD populations. As we identified only 26 studies over the last 14 years in this field, gaps surrounding provision of cancer survivorship care in CALD populations remain.

**Implications for cancer survivors:**

Ensuring culturally sensitive and specific delivery of cancer survivorship programs and interventions is paramount in providing optimal care for survivors from CALD backgrounds.

**Supplementary Information:**

The online version contains supplementary material available at 10.1007/s11764-023-01442-w.

## Introduction

In 2021, census data ascertained that there are over 45 million people living in the United States who are born overseas, of which over 25 million people speak English “less than very well”.^1^ Outside the United States, many other predominantly English-speaking countries such as Canada and Australia also have substantial immigrant populations.^2, 3^ Whilst there are various terms used to refer to these populations in a healthcare setting such as non-English speaking, English second language or limited English proficiency, in Australia they are recognised as Culturally and Linguistically Diverse (CALD), defined by the Australian Bureau of Statistics as people born overseas, in countries other than those classified by the Australian Bureau of Statistics as ‘main English-speaking countries’.^4^ This definition is more all-encompassing, acknowledging that differences in culture between native and non-native populations signpost complexities that are not captured when focusing purely on language as a point of difference.

Within healthcare, there is increasing recognition that CALD populations face disparities in disease management although they remain relatively poorly studied. For those with a cancer diagnosis, compared with dominant ethnic populations, being from a CALD background has been linked with poorer outcomes including more treatment toxicity, low survival and poorer quality of life.^5–7^ Potential barriers to optimal care among people from CALD backgrounds include inadequate resources to adjust for limited English proficiency and understanding of the local health system, low health literacy and poor communication with healthcare providers.^8–10^ As a consequence, lack of empowerment for medical decision-making, higher unmet needs and increased distress are prevalent amongst CALD populations with cancer.^11, 12^

The experience of patients with cancer can also be affected by communication difficulties and perceived discrimination, as demonstrated by a number of interview-based studies, which highlighted factors such as complexity of care needs, perceived discrimination, accessibility of care, and requiring assistance with understanding and navigating health systems to be serious concerns of patients undergoing anti-cancer treatments^13–15^. One scoping review of 60 qualitative and mixed-methods studies identified that major themes and issues CALD patients experienced aside from communication barriers included perceived lack of rapport with healthcare professionals, and cultural safety, including religious and ethno-cultural practices and beliefs.^15^

Across many cancer subtypes, significant improvements in survival have led to a paradigm shift in approaching cancer as a chronic illness. However, even after completion of primary treatment, long-term treatment toxicities, physical symptoms and psychosocial issues, including symptoms of anxiety and depression, and fear of cancer recurrence, can impact quality of life adversely.^16, 17^ As cancer survivors who have completed cancer treatment, these patients are often encouraged to self-manage their symptoms, schedules and medications which are often complex and multifaceted. Reviews of the literature have identified that although self-management and guided self-management support interventions have been studied in survivorship populations, outcomes were heterogeneous and benefits were temporary, suggesting that interventions should be tailored to the needs of specific populations.^18, 19^ Additionally, accommodating for priority populations in this context, such as CALD groups, was limited, indicating a requirement to focus on specific needs and outcomes of CALD populations.

The aim of this scoping review was to explore the breadth of international literature covering this understudied topic focused upon CALD populations in the survivorship setting.

## Methods

We developed a protocol using the scoping review methods proposed by Arksey and O’Malley^20^, refined by the Joanna Briggs Institute.^21^ This review was registered through the Open Science Framework.^22^ The PRISMA extension for scoping reviews^23^ was used to guide reporting.

### Eligibility criteria

Our eligibility criteria were defined using ‘Population, Intervention, Comparison, Outcomes, Study designs, Timeframe’ (PICOST) components.^24^ The population of interest was cancer survivors of adult-onset cancer who have completed curative intent treatment, who were identified as being from CALD backgrounds. For the purposes of this study, we define CALD populations as those from non-English speaking backgrounds, or people born outside of the non-native country whose first language is not English. Individuals generally self-identify as CALD. This definition does not encompass Indigenous or First Nations populations as they are not migrant populations, and the historical contexts of invasion and dispossession have given way to unique sociopolitical and cultural issues impacting healthcare in these specific populations. Interventions of interest were cancer survivorship programs or interventions; defined as healthcare services, usually multidisciplinary, aiming to improve the services and care for cancer survivors through research, education and/or an understanding of the issues that affect people who have been treated for cancer.^17^ Studies could be of qualitative, quantitative or mixed-method design, but had to have the full-text published in English. There were no restrictions on the types of primary outcomes studied for inclusion in the review. We excluded studies focused on: childhood cancer survivors; advanced/metastatic cancer survivorship; patients with haematological cancers; African-American populations; systematic and scoping reviews; and non-peer reviewed articles, conference abstracts, dissertations and commentaries.

### Information sources and search strategy

The protocol for the comprehensive search strategy was developed by LK in consultation with an Academic Liaison Librarian from the University of Sydney. The search was conducted by LK in Medline (Ovid), Embase, CINAHL, PsycInfo and Scopus from database inception to April 30, 2022. Grey literature was not searched as it was beyond the scope of this review to analyze non-indexed journal databases. The strategy for Medline is presented as Supplementary File 1. Additional search strategies are available from the corresponding author upon request.

### Study selection

Search results were imported into EndNote version 20 citation software; after initial checking the results were uploaded into Covidence for duplicate removal, two-tiered screening, and data extraction.

A series of calibration exercises prior to each stage of screening to ensure reliability across reviewers was completed. Inter-rater agreement for study inclusion was calculated using percent agreement and when it reached > 75% across the research team, we proceeded to the next stage. If the percent agreement was < 75%, the inclusion criteria were clarified, and another pilot test occurred. For abstract screening, one pilot test of 50 citations was conducted with all team members and we achieved 90% agreement. Subsequently, two reviewers (LK and WY) independently reviewed all titles and abstracts for inclusion. For full-text screening, one pilot test of 5 full-text articles was conducted, and we achieved 85% agreement. Following this calibration exercise, two reviewers (LK and WY) screened full-text articles for inclusion. All discrepancies between reviewers were resolved by consensus.

### Data collection

For each study, data were abstracted on study characteristics including study country, ethnic population of interest, cancer type, and languages used to deliver intervention. Other variables extracted included: study design; inclusion and exclusion criteria; method of participant recruitment; sample size (at time of screening and enrolment) and recruitment rate; study aims; baseline characteristics (including age, country of birth, time spent in country, employment status, marital status, years since diagnosis, education); disease and treatment characteristics (stage at diagnosis, rates of surgery, radiation, chemotherapy and hormonal therapy); study interventions (including mode of delivery, development process and cultural competence of staff delivering intervention); study endpoints and results; and dropout rates. Barriers and facilitators to survivorship care outlined or implied by authors were also recorded. The data abstraction form was developed and modified based on feedback from the study team. Data were extracted by two reviewers (LK and WY) and cross-checked by LK, with discrepancies resolved through consensus.

### Methodological quality appraisal

As this was a scoping review, quality appraisal and risk of bias of studies were not assessed.

### Data synthesis and charting

Extracted data were compiled into an Excel spreadsheet, from which descriptive statistical analyses were generated. Ethnic demographics of interest were categorised into Hispanic/Latina/Latino, Chinese, Asian-American or Other. For studies where more than one non-English language was used to deliver the intervention, this was recorded as single and aggregate values. Study designs were recorded as randomized controlled, non-randomized experimental, mixed-method or qualitative studies. For studies that listed initial screening sample sizes in addition to final enrolled sample sizes, the mean recruitment rate was calculated by dividing enrolled sample size by screening sample size. Dropout rates were calculated where data were available. Study endpoints were listed, and data were analysed qualitatively through content analysis to determine types of endpoints. Study interventions were listed in detail and sub-grouped as single- or multi-modal to reflect the number of components delivered, and content analyses were performed. Studies that met their primary endpoints were recorded, and for those that did not, those that met their secondary endpoints were recorded. Barriers and facilitators to cancer survivorship care were coded by reviewing manuscripts and were either identified explicitly by the authors, or implied in the results, and discussion sections of papers. Subsequent content analyses were performed and synthesized narratively, in addition to being grouped by frequency.

## Results

**Fig. 1:**
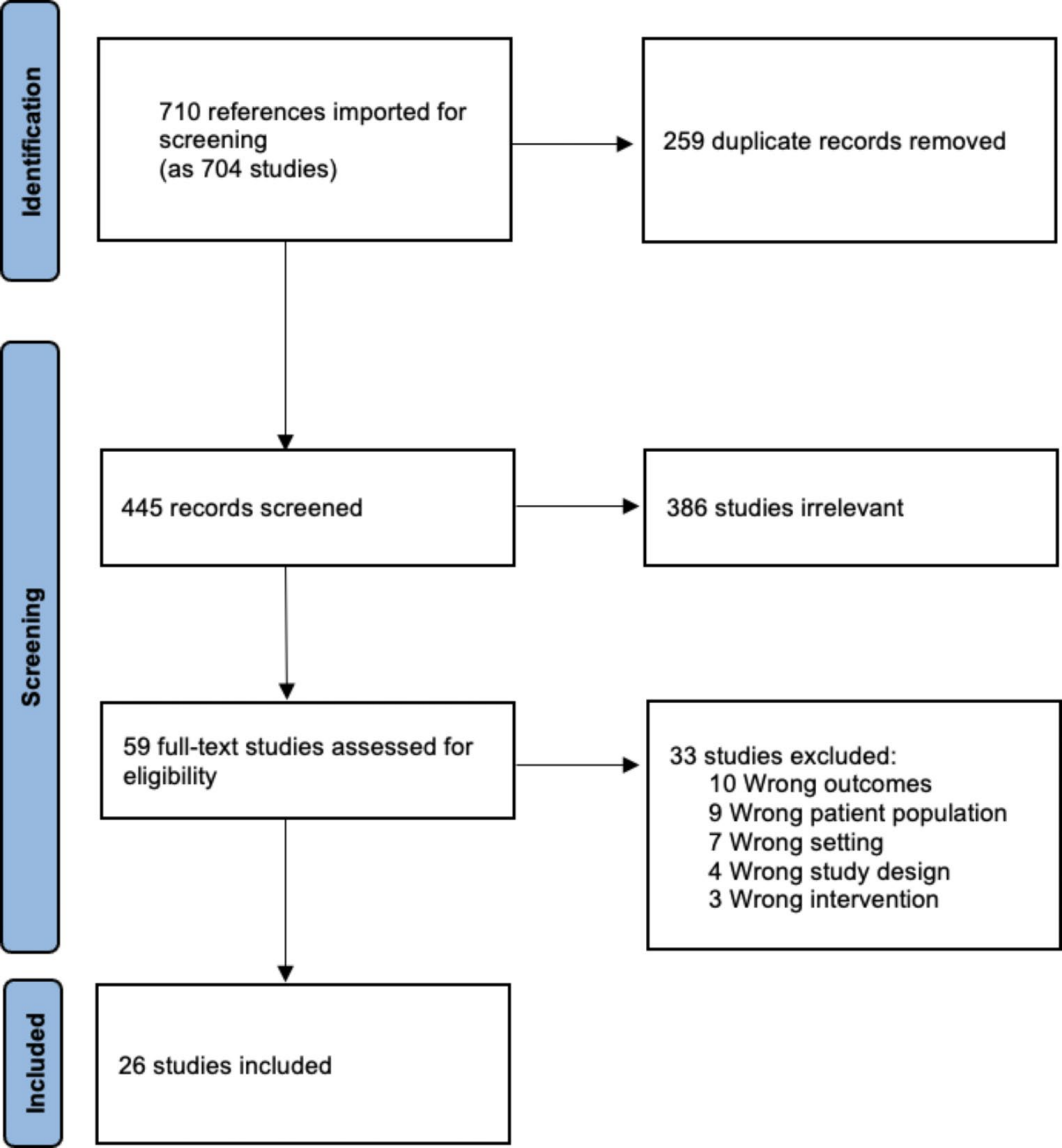
PRISMA-ScR flow diagram

The electronic database search obtained 710 results (Fig. 1): 259 duplicates were removed and 445 records underwent abstract screening. Overall, 59 were eligible for full-text review, and 26 were included for data extraction (Table 1). The full reference list is included in Supplementary Appendix.

**Table 1:** Summary of studies included for qualitative analysis

Primary author, Year, and Study country	Ethnic group of interest	Cancer type	Languages used to deliver intervention	Study design	N	Aim of study	Study intervention	Control arm (as applicable)	Primary endpoint(s)	Secondary endpoints	Results
Ashing, 2014 United States	Latina	Breast	Spanish, English	RCT	221	Test effectiveness of telephone psycho-educational intervention to reduce depressive symptoms	Survivorship education booklet; and eight 40-50-min, biweekly psycho-educational telephone sessions on: (i) breast cancer; (ii) managing medical issues and follow-up care; (iii) coping skills and problem solving; (iv) stress management; (v) social concerns; (vi) sexual health; and (vii) financial issues.	Survivorship education booklet: cancer information, psychosocial impact, and culturally sensitive resources on low-cost surveillance and treatment, medical, and psychosocial services	Differences in depressive symptoms (CES-D) across time within study condition	Differences in depressive symptoms: over time, within language group, by study arm, and combinations of the above	Decrease in depressive symptoms in the intervention (baseline M = 25.2; follow-up M = 17.2), vs. unchanged for control (baseline M = 14.8; follow-up M = 14.1). Change in mean depressive symptoms across time between groups was significant (95% CI: -5.75, -0.282; p < 0.05). Greater proportion of Latina BCS receiving intervention (63%) than control (26%) achieved 5 + point decrease in depressive symptoms at follow-up (adjusted odds ratio = 2.3; 95% CI: 1.1‚4.6; p < 0.05).
Ashing-Giwa 2008 United States	Latina-American	Cervical	Spanish, English	Non-randomized experimental study	23	Assess feasibility of telephone delivered, problem-focused, culturally-responsive intervention	Survivorship Kit; and Resources/counselling tailored to participant-initiated concerns. Domains: (1) orientation, (2) health education, cancer related resources and culturally appropriate referrals, (3) coping skills and problem solving, (4) stress management, (5) family communication, (6) relational and sexual communication, (7) social supports, and (8) contextual reinforcement.	“Survivorship Kit”: written information about cervical cancer (sexuality, stress management, communication with doctors and family, information on clinical trials, nutrition, and available resources)	FACT-G QoL		Significant improvement in physical well-being and overall QoL in intervention group. No improvement in social/family, emotional, and functional scores in either group. Post-intervention, percentage of women scoring below 50 on overall FACT-G score (range 0-100) decreased by half for both groups.
Buki 2021 United States	Latina	Breast	Spanish	Qualitative research	15	To examine perceptions of factors associated with effective delivery of psychosocial program designed for Spanish-speaking BCS; To identify mechanisms how program enhanced psychological well-being	Multidisciplinary, 3-session psychoeducational group intervention programme developed by investigators to address BCS issues	N/A	Qualitative – 15 open-ended questions assessing perceptions, feelings, and experiences related to psychosocial service provision		Effectively promoted access and enhanced women’s psychological well-being. 3 aspects contributed to sense of community: access factors, holistic approach to health promotion, and therapeutic factors imparted through support group. Access facilitated by services being free and in Spanish, offering a welcoming space, being near public transport, having an open-door policy. Perceptions of staff were that they were effective and highly skilled. Psychoeducational presentations and exercise classes were popular and satisfaction was high.
Buscemi 2019 United States	Hispanic	Breast	Spanish, English	Non-randomized experimental study	24	To evaluate feasibility of My Guide app for Hispanic BCS; to assess participant satisfaction with My Guide; and to report findings on the knowledge and preliminary intended effects (HRQoL) of My Guide	My Guide application: written/video/audio on enhancement of psychosocial adaptation after breast cancer, knowledge, stress management, social support, and communication. Additional weekly 15-min telecoaching calls from bilingual Spanish speakers to facilitate adherence.	N/A	Engagement – recruitment, retention, participant use	Acceptability – satisfaction survey, open ended feedback (post-trial) Knowledge – Knowledge about Breast Cancer Questionnaire (baseline and post trial) HRQoL – FACT-G7 (weekly)	Recruitment 75%, retention 91.7%. Mean hours use 9.25 h and telecoaching rate 93% (mean 2.8 calls out of 3). Mean satisfaction score 65.91 (range 42–70). Most common desires: information on diet and physical activity (45%), additional videos (32%), relaxation exercises (32%), content on cancer recurrence (27%). Breast Cancer Knowledge Questionnaire improved from baseline (M = 9.50, SD = 2.92) to post-trial (M = 11.14. SD = 2.66); t(21) = -3.12, p = 0.002, d = 0.59 (medium effect size). No statistically significant improvement in FACT-G7 scores.
Chan 2017 Other: Singapore	Asian (85% Chinese, 8% Malay, 5% Indian)	Breast	English	RCT	72	To evaluate the effects in Asian BCS on distress reduction in physical and psychological symptoms receiving psychoeducational programme compared with usual care	Multidisciplinary psychoeducational programme – 3 educational sessions, 4.5 h each. Conducted by health-care professionals addressing concerns endorsed in guidelines for quality cancer survivorship published by the American Institute of Medicine (IOM). Guidelines culturally adapted e.g. Chinese medications, Asian dietary preferences. Trainers focused on impact of BCS specific problems and coping strategies.	Usual care – provided with an information booklet on self-management of cancer, and treatment-related symptoms.	Mean change in the RSCL physical symptom distress scores and psychological distress scores for each group	Mean change in total BAI score, mean change in EORTC QLQ-C30 functional scale scores, and descriptive responses from the patient satisfaction questionnaire	Intervention had greater reduction of physical symptom distress over time than control (-2.1 ± 7.4 vs. -8.2 ± 9.4 points; P < 0.01), Cohen d-effect size 0.72. Change of psychological distress similar between groups (-1.6 ± 15.6 vs. -4.0 ± 18.8 points;P = 0.55). Both groups had improved overall valuation of life over time. QLQ-C30 and BAI scores not statistically significant between groups or over time. Satisfaction questionnaire (response rate 82%) – all agreed content taught addressed needs in dealing with survivorship issues.
Chee 2017 United States	Asian American	Breast	English, Mandarin, Korean, Japanese	RCT	65	To qualitatively evaluate Internet Cancer Support Group for Asian Americans (ICSG-AA) through usability test and expert review; to evaluate efficacy of ICSG-AA in enhancing outcomes	ICSG-AA – theory-driven culturally tailored internet cancer support group for Asian American BCS	Links to internet resources related to Asian Americans and daily life	Changes in: Perceived social support (PRQ) Perceived interactions (PIS) Support care needs (SCNS-SF34) Uncertainty (MUIS-C) Self-efficacy (CBI) Physical/psychological symptoms (MSAS-SF) Pain (BPI) QoL (FACT-Breast)		Improvement in support care needs (F = 22676.20, p < 0.01) and physical and psychological symptoms (F = 309.11, p < 0.05) of control group from pre- to post-test. Decrease in uncertainty of intervention (F = 127.30, p < 0.10). Intervention group had greater improvements in physical and psychological symptoms (F = 3.16, p < 0.10) and QoL (F = 3.31, p < 0.10).
Chee 2020 United States	Asian American	Breast	English, Mandarin, Korean, Japanese	RCT	94	To determine preliminary efficacy of the technology-based Cancer Pain management support Program for Asian American survivors of breast cancer (CAPAA) in improving the cancer pain experience of Asian American BCS	CAPAA: (1) interactive online discussion board with daily moderating, coaching and monitoring by culturally matched registered nurses under the supervision of culturally matched doctors; (2) online educational sessions – culturally sensitive information about BCS-related topics and cancer pain management (tested and validated in prior studies); (3) language/culture specific BCS-related internet links	American Cancer Society website on breast cancer and cancer pain management	Difference in degree of cancer pain (BPI-SF)	Support care needs – SCNS-34SF Uncertainty – MUIS-C Personal resources – PRQ Perceived isolation – PIS Perceived self-efficacy – CBI self-efficacy items	No statistically significant differences in pain. Improvements in perceived isolation (F = 9.937, P < 0.01), personal resources (F = 6.612, P < 0.05), support care needs (F = 8.299, P < 0.01), and degree of uncertainty (F = 8.722, P < 0.01) in the intervention group from pre- to post-test. Perceived isolation improved more in intervention (mean = -0.43, SD = 0.79) than control group (mean = 0.11, SD = 0.20; F = 5.471, P < 0.05). Personal resources increased in intervention (mean = 0.53, SD = 0.59), and decreased in the control group (mean = -0.11,SD = 0.68; F = 10.027, P < 0.01). Uncertainty decreased in intervention (mean =-0.49, SD = 0.87), and increased in control group (mean = 0.05, SD = 0.37; F = 4.455, P < 0.05).
Chu 2019 United States	Chinese American	Breast	Mandarin, Cantonese	RCT	96	To investigate how the efficacy of expressive writing on different PTSD symptom clusters (re-experiencing, avoidance, and arousal) would vary depending on acculturation	Self-regulation (S) arm: Write about deepest feelings and thoughts related to their breast cancer experience at week one, their coping strategies to deal with stressors caused by breast cancer at week two, and positive thoughts and feelings regarding their breast cancer experience at week three	Emotional disclosure I arm: Write about deepest thoughts and feelings about their cancer experience weekly, for three weeks Cancer-fact I arm: Write about cancer diagnosis and treatment as objectively and detailed as possible weekly, for three weeks	Change in PTSD symptoms: PTSD Symptom Scale-Self Report (PSS-SR) and DSM-IV-TR criteria - re-experiencing, avoidance, arousal	Manipulation check (Likert scale): how personal writings were, how much revealed emotion, and how much increased understanding about disease Acculturation (as moderator): Stephenson Multigroup Acculturation Scale-Dominant Society Immersion Subscale	Re-experiencing: acculturation moderated writing effect (E vs. S) at 6-month follow-up (β = -0.23, p = 0.041). Low acculturation (LA) S participants lower re-experiencing than E (p = 0.038); for high acculturation (HA) no difference between groups (p = 0.432) Avoidance: acculturation moderated writing (E vs. C) at 6-months (β = -0.38, p = 0.012). LA C had lower avoidance than E (p = 0.010); for HA, no difference between groups. Arousal: acculturation moderated writing (E vs. C) at 3-months (β = -0.31,p = 0.030) and 6-months (β = -0.35,p = 0.014) and the writing (E vs. S) at the 6-months (β= -0.30,p = 0.02). At 3 months, LA C group had lower arousal than E (p = 0.023); for HA participants, there was no difference. At 6 months, C (p = 0.001) and S group (p = 0.016) LA participants had lower arousal than E group; HA participants showed no differences (ps > 0.10). No effects of acculturation on PTSD”symp’om clusters (ps > 0.100).
Dieli-Conwright 2019 United States	Hispanic, non-Hispanic	Breast	Spanish, English	RCT	97	To explore whether Hispanic BCS will have poorer physical fitness and QoL, and may derive greater benefits from exercise than non-Hispanic BCS	16-week exercise program aligned with American College of Sports Medicine/American Cancer Society exercise guidelines for BCS. 3 supervised, 1-on-1 weekly exercise sessions including aerobic and resistance exercises.	Log/maintain current level of physical activity throughout the 16-week study period. At study completion, participants were offered the identical exercise intervention	Change in metabolic syndrome (MSY) z score (calculated from modified z scores: waist circumference, systolic blood pressure/diastolic blood pressure, high-density lipoprotein cholesterol (HDL-C), triglycerides (TG), and glucose)	Physical activity assessment (questionnaires) Dietary assessment Medical history (Charlson Comorbidity Index) Physical fitness (treadmill test, estimated maximal oxygen uptake; maximal voluntary strength) PROs – FACT-B (breast cancer specific QoL), SF-36 (global health status), CES-D (risk for depression)	Baseline – 85% Hispanic BCS and 69% Non-Hispanic BCS had MSY. Intervention group improved in all MSY variables compared with baseline (P < 0.01) and usual care (P < 0.001). Effect maintained at 28 weeks. Ethnicity moderated effect of exercise on HDL-C (11.3 mg/dL; 95% CI, 18.7 to 6.2 mg/dL), TG (-36.4 mg/dL; 95% CI, -64.1 to -18.8 mg/dL), glucose (-8.6 mg/dL; 95% CI, -19.1 to -3.0 mg/dL), and MSY z scores (-3.0; 95% CI, -7.0 to -0.8). Globally, Hispanic had greater improvements vs. Non-Hispanic BCS.
Elimimian 2020 United States	Hispanic	Breast	Spanish, English	Non-randomized experimental study	94	To evaluate long-term benefit of implementing language-appropriate, Mindfulness-Based Stress Reduction (MBSR) program targeting Hispanic BCS	MBSR program – 8 week, 2-hr/week course delivered by bilingual psychologist,. Components: teaching mindfulness and practicing techniques in class, home practice through audio recordings, daily diary writing, and a book on mindfulness.	N/A	Changes in: Anxiety – GAD7 Depression – PHQ9 Physical QoL – SF-36-PCS Mental QoL – SF-36-MCS		Improvements in GAD7 (mean change = -2.39, P = 0.04) and PHQ9 scores (mean change = -2.27, P = 0.04) at 24 months compared with baseline. For each outcome, improvement in PHQ9 at 24 months was 15.2%, indicating persistence of benefit, Longitudinal effects: improvements in all measures except PCS at 12 and 24 months; mean change in MCS 4.07 (95% CI = 0.48 to 7.66, P = 0.03), GAD7 -2.66 (95% CI = -4.65 to -0.67, P = 0.01), and PHQ9 -3.01 (95% CI = -4.87 to -1.16, P < 0.001).
Greenlee 2015 United States	Hispanic	Breast	Spanish	RCT	70	To examine effects of culturally-based approach to dietary change on increasing fruit and vegetable (F/V) intake, in BCS over 6 months	9 session (24 h, 12 weeks) nutrition intervention program focused on achieving and maintaining nutrition-related guidelines of American Cancer Society and American Institute for Cancer Research. All also received control booklet	22-page Spanish-language booklet on healthy eating for breast cancer survivors	F/V intake, fat intake	Anthropometric measurements (weight, body measurements)	Intervention compared with Controls reported increase in mean servings of F/V per day (all F/V: +1.1 vs. -0.3; p = 0.05; targeted F/V: +2.0 vs. -0.2; p = 0.004) at 3 months; changes maintained at 6 months. At 6 months, there was a non-significant trend in difference in % weight change between groups. Significant difference in waist circumference between groups at 6 months; Intervention group mean − 1.6 cm, Control arm + 1.7 cm (P = 0.05).
Im 2019 United States	Asian American (Chinese, Japanese, Korean)	Breast	English, Mandarin, Korean, Japanese	RCT	91	To evaluate impact of Technology-Based Information and Coaching Program for Asian American BCS (TICAA) compared with American Cancer Society (ACS) Website in self-reported menopausal symptoms over 1 and 3 months	TICAA: Theory-driven and culturally tailored intervention program, aiming to provide information and coaching/support for Asian American BCS, containing language- and culture-specific education and resources, with group/individual coaching by culturally-matched nurse interventionists. All participants had access to control ACS website	ACS website	Change over time in: Menopausal symptoms distress and frequency – MSAS-SF	Attitudes and social influences – Questions on Attitudes, Subjective Norm, Perceived Behavioral Control, and Behavioral Intention Perceived Barriers – modified Barriers to Health Activities Scale Self efficacy – CBI-B	Intervention group – decrease in distress of menopausal symptoms over time: physical (b = 0.07, P = 0.08), psychological (b = 0.13,P = 0.05), psychosomatic (b = 0.17, P = 0.6), total symptoms (b = 0.19, P = 0.0). Time effect was not statistically significant. Frequencies of psychological symptoms decreased over time for both groups (b = 0.11, P = 0.01) but time-study arm interaction not significant.
Im 2020 United States	Asian American (Chinese, Japanese, Korean)	Breast	English, Mandarin, Korean, Japanese	RCT	115	To evaluate impact of Technology-Based Information and Coaching Program for Asian American BCS (TICAA) compared with American Cancer Society (ACS) Website with regard to self-reported pain and accompanying symptoms over 1 and 3 months.	TICAA: Theory-driven and culturally tailored intervention program, aiming to provide information and coaching/support for Asian American BCS, containing language- and culture-specific education and resources, with group/individual coaching by culturally-matched nurse interventionists. All participants had access to control ACS website	ACS website	Change over time in: Pain/symptom distress – MSAS-SF	Attitudes – QASPB attitudes subscale Self-efficacy – CBI Perceived barriers – QASPB perceived barriers subscale Social influences – QASPB social influences subscale, PRQ-2000, PIS	Both groups demonstrated significant decreases in all outcome variables (frequencies and distress scores of pain and the distress scores of global, physical, and psychological symptoms) over time. Greater decreases in physical/psychological symptom distress scores in intervention compared with control, especially month 1 (β= -0.22 [P = 0.0062] for physical and β= -0.18 [P = 0.0319] for psychological). Decrease in perceived barriers scores noted in intervention but not control group (β= -3.55 [P = 0.0033]).
Im 2021 United States	Asian American	Breast	English, Mandarin, Korean, Japanese	RCT	67	To evaluate impact of Technology-Based Information and Coaching Program for Asian American BCS (TICAA) compared with American Cancer Society (ACS) Website in improving self-efficacy for coping with breast cancer.	TICAA: Theory-driven and culturally tailored intervention program, aiming to provide information and coaching/support for Asian American BCS, containing language- and culture-specific education and resources, with group/individual coaching by culturally-matched nurse interventionists. All participants had access to control ACS website	ACS website	Self-efficacy – CBI-B	Perceived social support – PRQ-2000 Perceived isolation – PIS Support needs – SCNS-SF34	Significant increases in self-efficacy of both groups over time. Significant increases in self-efficacy scores of intervention group from pre- (81.12 ± 25.61) to 1 month (86.82 ± 19.59) and 3 months (86.82 ± 19.60). Increase in control group’s self-efficacy was only at 1 month (80.697 ± 21.486), with decrease at 3 months (79.848 ± 20.048). Subgroup analysis: TICAA more effective if high needs (patient care, support), low-education and low perceived social support; and in those with high needs who were in the high-education group, aged over 40.5 years, with low social support.
Juarez 2013 United States	Latina	Breast	Spanish, English	RCT	52	To test the effectiveness of an English/Spanish education intervention to assist Latina BCS transition into survivorship	Nueva Luz: individualized bilingual program designed to provide Latinas with breast cancer with structured, culturally and linguistically tailored information about common QoL concerns and strategies to assist transition into survivorship. 4 Sessions: (1) Physical Well-Being; (2) Psychological Well-Being; (3) Social Well-Being (4) Spiritual Well-Being.	Usual care (supportive care services, monthly educational workshops, support groups, access to a cancer information resource nurse, written materials through resource center), monthly telephone calls.	Change in means in uncertainty (MUIS-C), distress (Psychological Distress Thermometer) and quality of life (City of Hope Quality of Life Breast Cancer questionnaire) between groups		Intervention and control arms had decrease in uncertainty (57.81 to 55.41 vs. 52.72 to 50.4 over 6 months), but not statistically significant controlling for acculturation. No change in means of distress scores between groups over time (p = 0.305). QoL did not show significant changes between groups or over time.
Kwok 2011 Australia	Chinese	Breast	Cantonese	Mixed-method study	29	To develop, and evaluate support group programme effectiveness (especially psychosocial and informational needs of the participants)	Culturally sensitive support group programme, delivered over 8 weeks (2 h/week) conducted in a Chinese cancer support organisation. Education re: management of breast cancer and group sharing. Chinese Medicine and Western health promotion strategies were discussed. Written Chinese information provided.	N/A	Evaluation form and Qualitative assessment (semi-structured interview)		Feedback: All appreciated cultural and linguistic sensitivity Informational effects: very relevant and supportive to needs (including linguistic and cultural) Psychosocial impact: sense of interconnectedness with others in similar situations, alleviating isolation. Also having group members of the same culture and language made participants more willing to express feelings and concerns.
Kwong 2016 Canada	Chinese	Any	Mandarin, Cantonese, English	Qualitative research; Other: Implementation research	123	To evaluate the impact of a structured psycho-educational program targeting Chinese cancer survivors (BE ACTIVE)	6 educational presentations (2 h/week) with handouts	N/A	Assess participants’ understanding of the material, motivation for change, and satisfaction with the sessions.		Highly useful and easy to understand. Very pleased with guest speakers knowledge, clear presentations, and ability to answer questions. High satisfaction overall – learning gains (6.17/7.0 in 2012 and 6.34 /7.0 in 2013). Highly recommended to other survivors (6.49/7.0 in 2012 and 6.67/7.0 in 2013). Qualitative feedback overall positive.
Le 2019 United States	Chinese American, Non-Hispanic White	Breast	English, Mandarin, Cantonese	Mixed-method study	397	To evaluate whether Chinese American BCS’ acculturation affected adherence to American Cancer Society (ACS) physical activity (PA) recommendations compared with non-Hispanic White (NHW) after controlling for covariates	No Intervention – ACS PA recommendations	N/A	Adherence to PA moderated by acculturation – IPAQ-SF	Anderson et al. short English acculturation survey to assess Chinese immigrants’ English proficiency in listening, speaking, reading, and writing (Likert scale) - MAC = interviewed in English or had good English proficiency (each proficiency score ≥ 4) AND lived in the U.S. ≥25 years (median) - LAC = not meeting above criteria	> 73% adherence: 80% NHW, 76% MAC and 60% LAC (p < 0.001). LAC least likely to do vigorous activity compared to MAC and NHWs (p < 0.001). LAC less likely to meet recommendations compared to NHW (OR 0.38, 95%CI 0.19–0.75, p < 0.05); no significant difference between MAC and NHW. Chinese BCS with low English proficiency or were interviewed in Chinese had lower adherence than NHW. Chinese immigrant groups had lower adherence rates than NHW regardless of duration of US residency.
Lu 2012 United States	Chinese	Breast	Mandarin, Cantonese	Mixed-method study	21	To evaluate whether expressive writing is associated with improvement in QoL, physical health and psychological adjustment	Write for 20 min weekly for 3 weeks about feelings and thoughts, coping strategies, and positive thoughts and feelings regarding experience with breast cancer	N/A	Change in scores: Quality of life – FACT-B Fatigue – FACIT-F Physical symptoms – Physical Symptom Checklist Affect – PANAS Posttraumatic growth – PTGI PTSD – PTSD Symptom Scale Cancer-related intrusive thoughts – Impact of Events Scale – Intrusion subscale	Semi-structured phone interview	Medium-large effects on the decrease of fatigue (0.066) and intrusive thoughts (0.124). Large effect on reducing posttraumatic stress at 3 months (0.208). Medium to large effects on the decrease of fatigue (0.066) and post-traumatic stress (0.1), and increase of QoL (0.087) and positive affect (0.099) at 6 months. Interviews: positive feedback about the study overall, commented that the study was meaningful for Chinese women.
Lu 2014 United States	Chinese (Mandarin, Cantonese)	Breast	Mandarin, Cantonese	Mixed-method study	15	To evaluate feasibility, cultural sensitivity, and effectiveness of a peer mentoring and educational intervention (Joy Luck Academy, JLA) designed for Chinese American BCS	JLA: 10 weekly sessions lasting 2 h each. Ate healthy meal with mentors, followed by educational sessions.	N/A	Change in depression and anxiety over time – BSI depression/anxiety subscales	Health outcomes questionnaire package (pre and post intervention) Process evaluation questionnaires – perceived satisfaction, usefulness, and appropriateness Focus group interviews – for mentors and mentees	Decrease in depressive symptoms pre- to post-test (t = 2.54, p = 0.03, d = 0.55). Anxiety decreased but not statistically significant. Process evaluation questionnaire: course very useful, high level of support from mentors, peers, and program facilitator. Focus groups: JLA was culturally sensitive, high satisfaction. Participants valued: education; in-depth discussion with health professionals; interactions with mentors; and discussion time. All mentee and mentor participants indicated that mentees were happier and more energetic.
Napoles 2015 United States	Latina	Breast	Spanish	RCT	151	To examine the Nuevo Amanecer (NA) program effectiveness in improving HRQoL and distress at 3 and 6 months Substudy: impact of mediators (emotional support, acceptance, fatalism) on emotional well-being	NA: cognitive behavioral stress management program aimed to increase self-efficacy for cancer coping, use of coping skills, and perceived social support. 8 weekly modules delivered face-to-face in participants’ homes.	Usual care; wait list control	Difference in change in QoL between groups – FACT-B	General distress – BSI Breast cancer specific distress – Intrusive Thoughts Scale Substudy: Emotional support – Medical Outcomes Study Social Support Survey Fatalism – Powe Fatalism Inventory Acceptance – Benefit Finding Scale Emotional well-being – FACT-QoL	At 3 months: intervention improved significantly vs. control on QoL/distress: emotional well-being, + 3.86 vs. + 1.87 points; enjoyment of life, + 0.78 vs. -0.50 points; somatization, -0.26 vs. -0.01 points. At 6 months, effects maintained and additional improvement in physical well-being, + 4.15 vs. + 1.68 points; breast cancer concerns, + 4.79 vs. + 2.59 points; overall QoL, + 14.18 vs. + 8.19 points; depression, -0.55 vs. -0.29 points. Substudy: At baseline, emotional support negatively associated with fatalism and positively with emotional well-being. Fatalism mediated this, whereas acceptance mediated only marginally.
Napoles 2020 United States	Latina	Breast	Spanish	RCT	153	To adapt and improve the generalizability of Nuevo Amanecer to be appropriate for rural, low literacy Spanish-speaking Latina BCS in improving QoL	Nuevo Amanecer-II (NA-II): 10 weekly modules for 90 min at participants’ homes: (1) managing impact; (2) breast cancer and survivorship; (3) cancer information; (4) support; (5) helpful and unhelpful thoughts; (6) thoughts/mood; (7) stress management; (8) managing activities that affect mood; (9) healthy lifestyles; (10) goal-setting. Written manual in Spanish	Wait-list control group	Difference in change in BC specific QoL between groups – FACT-B	Depression – PHQ-8 Perceived stress – 10-item PSS Spanish version Anxiety and somatization – two scales from BSI Stress management – MOCS-A	No statistically significant treatment by time interaction effects on QoL at 3 or 6 months. At 6 months treatment by time improved anxiety in intervention vs. control (P = 0.049; -0.20 vs. -0.02). At 6 months, somatization in intervention lower than control (0.48 vs. 0.65; range 0–4;P < 0.05). No significant treatment by time interaction effects for somatization, depression or stress. Stress management: significant treatment by time interaction effects for Relaxation, awareness of tension, and coping confidence
Napoles 2019 United States	Latina	Breast	Spanish	Mixed-method study	23	To develop and evaluate feasibility, acceptability, and preliminary efficacy of Nuevo Amanecer (NA) Survivorship Care Planning (SCP) program for Spanish-speaking breast cancer patients treatment	NA: 2-month intervention comprising 4 components: (1) individualized bilingual SCP, (2) Spanish-language information booklet, (3) Spanish-language mobile app (trackC) with integrated activity tracker (Fitbit Zip), and (4) 5 weekly health coaching telephone calls in Spanish to reinforce survivorship care concepts and positive health behaviors.	N/A	Symptom PROM: PROMIS Cancer-Fatigue Scale Medical Outcomes Study Health Distress Scale Knowledge PROM: Self-efficacy PROM: 8-item self-efficacy for managing cancer scale	FACT-G emotional wellbeing scale Depression – PHQ-8 BSI Somatization Scale Average daily steps Debriefing interviews Satisfaction survey	Fatigue (B= -0.26; P = 0.02; Cohen d = 0.4) and health distress (B= -0.36; P = 0.01; Cohen d = 0.3) lower post-intervention. Greater knowledge of recommended follow-up care and resources after intervention (B = 0.41; P = 0.03; Cohen d = 0.5); self-efficacy unchanged. Emotional well-being and average daily steps significantly improved (B = 1.42; P = 0.02; Cohen d = 0.3). Average daily steps increased significantly. 91% completed satisfaction surveys, with quality, ease of use and usefulness reported as quite or very useful in most. Debriefing interviews (n = 10) – generally positive – felt supported by the app and weekly check-ins, and understood disease and symptoms better. App- easy to use but issues with support at first, or due to lower literacy. Enhanced physical and emotional well-being.
Warmoth 2020 United States	Chinese	Breast	Mandarin, Cantonese	Non-randomized experimental study	43	To investigate the potential psychological benefits of the Joy Luck Academy (JLA) psychosocial intervention for Chinese BCS	JLA: 10 weekly sessions lasting 2 h each. Ate healthy meal with mentors, followed by educational sessions.	N/A	Positive affect – PANAS Post-traumatic growth – PTGI		Significant improvement in positive affect (d = 0.7) after intervention. Overall PTGI (d = 0.51) and Relating to Others subscale (d = 0.55) improved with medium effect sizes. Appreciation for Life subscale demonstrated large effect size improvement (d = 0.94).
Wiley 2018 Australia	Arabic, Italian, Vietnamese	Any	English, Arabic, Italian, Vietnamese	Mixed-method study	39	To develop evidence-based, culturally appropriate, consumer-informed written resources providing information and education on what to expect when completing cancer treatment	Written information and education on cancer treatment processes for Arabic, Italian and Vietnamese cancer survivors	N/A	Development of culturally sensitive written resources in conjunction with consumer and stakeholder input	Focus groups/qualitative	Lessons learned in processes of resource development: Community engagement and consultation; Culturally sensitive data collection; Trained focus group facilitators; Culturally and linguistically specific content development with editorial input to ensure readability; Translation and review of design and format by consumers; Dissemination and sustainability – focus group results suggest in-language radio or print media distribution through health providers or community groups, with regular updates. Complete outcomes data to be presented in separate paper
Yanez 2020 United Statesnter	Latina	Breast	Spanish, English	RCT	80	To establish the feasibility and preliminary efficacy of My Guide smartphone app compared with My Health app	My Guide application – written/video/audio focused on enhancement of psychosocial adaptation in BCS, breast cancer-related knowledge, coping with side effects, stress management, social support, and communication. Additional, 15-min motivational telecoaching calls and texts from bilingual native Spanish speakers to facilitate adherence	My Health attention-control app - recommendations for nutrition, physical activity, prevention of chronic illness, other healthy lifestyle behaviors. Schedule similar to experimental arm	Feasibility across timepoints (baseline, post-intervention and 2 weeks post) - recruitment (70%) - retention (80%) - usage (90 min per week)	Acceptability – HRQoL – FACT-B Symptom burden – BCPT Secondary analysis: App use HRQoL – FACT-G7 Cancer specific distress – Impact of Events scale Cancer Relevant Self Efficacy – CASE-cancer Breast Cancer knowledge – Knowledge about Breast Cancer questionnaire	Recruitment 79%, retention > 90%. Proportion who met the threshold of using their assigned app did not differ between groups (P = 0.247). 97% of My Guide and 92% of My Health were satisfied with app (agree or somewhat agree; χ2 = 5.41, P = 0.144). 100% of My Guide and 95% of My Health would recommend their assigned app to another BCS (χ2 = 2.00, P = 0.157). 92% My Guide and 84% My Health reported they would like to continue using the app (agree or somewhat agree; χ2 = 4.59, P = 0.332). No Interaction of time and condition on symptom burden or breast cancer well-being.

Abbreviations: RCT = randomised controlled trial; BCS = breast cancer survivor; FACT-G = functional assessment of cancer therapy – general; QoL = quality of life; CI = confidence interval; CES-D = center for epidemiologic studies depression scale; HRQoL = health-related quality of life; FACT-G7 = functional assessment of cancer therapy – general – 7 item scale; RSCL = Rotterdam Symptom Checklist; BAI = Beck anxiety inventory; EORTC-QLQ-C30 = European organisation for research and treatment of cancer – quality of life questionnaire – C30; PRQ = personal resource questionnaire; SCNS-SF34 = supportive care needs survey – short form 34 item; MUIS-C = Mishel uncertainty in illness scale – community; CBI = cancer behaviour inventory; MSAS-SF = Memorial symptom assessment scale – short form; BPI = brief pain inventory; FACT-B = functional assessment of cancer therapy – breast; BPI-SF = brief pain inventory – short form; PTSD = post-traumatic stress disorder; PSS-SR = PTSD symptom scale – self-report; SD = standard deviation; SF-36 = medical outcomes study questionnaire short form – 36 item; GAD = general anxiety disorder; PHQ9 = patient health questionnaire-9; IPAQ-SF = international physical activity questionnaire – short form; MAC = more acculturated; LAC = less acculturated; FACIT-F = functional assessment of chronic illness therapy – fatigue; PANAS = positive and negative affect schedule; PTGI = post-traumatic growth index; BSI = brief symptom inventory; MOCS-A = measure of current status – part A; PROMIS = patient reported outcome measures information system; BCPT = breast cancer prevention trial symptom scale; CASE-cancer = communication and attitudinal self-efficacy scale for cancer

### Demographics

Most studies were conducted in the United States (85%) and focused on breast cancer survivors (88%; Table 2). The most studied ethnic demographic was Hispanic/Latino/Latina, (54%) followed by Chinese (27%) and “Asian-American” (19%, which encompassed Chinese, Japanese and Korean). In total, 96% of programs were delivered in non-Native languages, including Spanish (46%) and Chinese (Mandarin or Cantonese, 27%).

**Table 2:** Characteristics of studies included in final scoping review analysis (n = 26)

Study Characteristic			N (%)
**Study Design**			
	Randomized trial		14 (54)
	Mixed-method		6 (23)
	Non-randomized experimental study		4 (15)
	Qualitative		2 (8)
**Study Country**			
	United States		22 (85)
	Australia		2 (8)
	Canada		1 (4)
	Singapore		1 (4)
**Cancer Type**			
	Breast		23 (88)
	Any solid		2 (8)
	Other solid		1 (4)
**Ethnicities Included**			
	Hispanic/Latinx		14 (54)
	Chinese		7 (27)
	Asian American		5 (19)
	Other		2 (8)
**Languages Used in Delivery**			
	Spanish		12 (46)
	Mandarin and/or Cantonese		7 (27)
	Mandarin, Japanese and Korean		5 (19)
	Cantonese only		1 (4)
	English only		1 (4)
	Arabic, Italian and Vietnamese		1 (4)
**Recruitment Method**			
	Voluntary/Community		13 (50)
	Clinic-based		9 (35)
	Mail		3 (12)
	Telephone		2 (8)
	Not stated		1 (4)
**Primary Endpoint Measures**			
	Patient-reported outcomes		17 (65)
	Qualitative		3 (15)
	Study specific questionnaire		2 (8)
	Clinical		2 (8)
	Feasibility		2 (8)
**Intervention**			
	Single modal (n = 5)	Written	5 (100)
		In-person	0 (0)
		Phone	0 (0)
		Online	1 (20)
	Multi-modal (n = 21)	Written	19 (90)
		In-person	13 (62)
		Phone	9 (43)
		Online	5 (24)
	Bilingual delivery (n = 24)	Yes	23 (88)
		No	1 (4)
		N/A	2 (8)
	Staff Training (n = 24)	Yes	13 (50)
		Not mentioned	11 (42)
		N/A	2 (8)
	Met primary endpoint (n = 22)	Yes	17 (77)
		No	5 (23)

### Study designs and recruitment

In terms of study design, there were 14 randomized (54%), 6 mixed-method (23%), 4 non-randomized experimental (15%), and 2 qualitative studies (8%). All had clearly defined eligibility criteria for inclusion. Half (50%) the studies recruited participants through voluntary participation from community-based centres or advertising, followed by clinic patients (34%), mail (12%) and telephone (8%). Thirteen (50%) studies provided data on initial screening sample sizes in addition to final enrolled sample sizes, with median recruitment rate 74% (mean 62%, range 23 to 91%). Overall median sample size was 71 (mean 87, range 15 to 397). Of the 18 studies that provided information about attrition, the median dropout rate was 10% with various cited reasons such as *withdrawal from intervention, loss to follow-up, lack of time or not feeling well*.

### Study interventions

There were several active research groups publishing in the domain of CALD survivorship with numerous papers listed overlapping authorship (Table 1), with 9 (35%) study interventions developed based on prior work by the same research group. These groups were all United States-based, and primarily focused on Spanish-speaking^25–29^ or Asian-American populations^30–38^. Twelve (46%) cited community-based participatory research methods or at least involvement of community stakeholder consultation in developing their respective interventions, and 12 (46%) were led by a steering committee. Six (23%) study interventions were developed from existing guidelines or programs. Interventions were delivered by a variety of staff including nurses (38%), doctors (31%), research staff (19%), psychologists (23%), other cancer survivors (19%), and exercise physiologists (19%). Only one (4%) of the studied interventions was fully self-administered. Staff training on cultural competence (where relevant) was mentioned in 13 studies (50%), and 23 (88%) of the studies utilized bilingual staff.

Most studies (n = 21, 81%) examined interventions with multiple components, and most delivered interventions through several avenues, including in-person, written, online, app and telephone delivery (see Table 2). Intervention program content was most focused around psychosocial domains (85%) or education about cancer (77%), with fewer studies focusing on other physical domains such as medication management (12%), exercise (12%) and diet (8%). Modes of intervention delivery included education (50%), coaching (35%), classes (12%), mentorship (8%), and through expressive writing done by participants (8%).

### Endpoints and results

Most studies utilized validated patient-reported outcome measures (PROMs), frequently as primary endpoints (n = 17, 65%), or secondary endpoints (n = 4, 15%). Of the other quantitative studies, primary endpoints studied included study-specific questionnaires (n = 3, 12%), feasibility (n = 3, 12%), and clinical-based endpoints (n = 2, 8%). Three (12%) studies were primarily qualitative in nature. PROMs encompassed a variety of variables, most commonly quality of life (46%), self-efficacy (27%), depression (23%), symptoms (19%), and anxiety, support care needs, satisfaction, and social factors (15% each). The two studies with clinical primary endpoints both examined the effects of diet or exercise interventions on Latina American breast cancer survivors.

Out of 23 quantitative studies, 17 (77%) met their primary endpoints (13 PROM, 2 feasibility, 2 clinical-based), and of the studies that did not meet their primary endpoints, 4 met at least one secondary endpoint (all PROMs). Only one study did not meet any of their endpoints. The most commonly studied PROM endpoint, quality of life, was significantly improved in 5 (42%) out of 12 studies.

### Barriers and facilitators to survivorship care

**Fig. 2:**
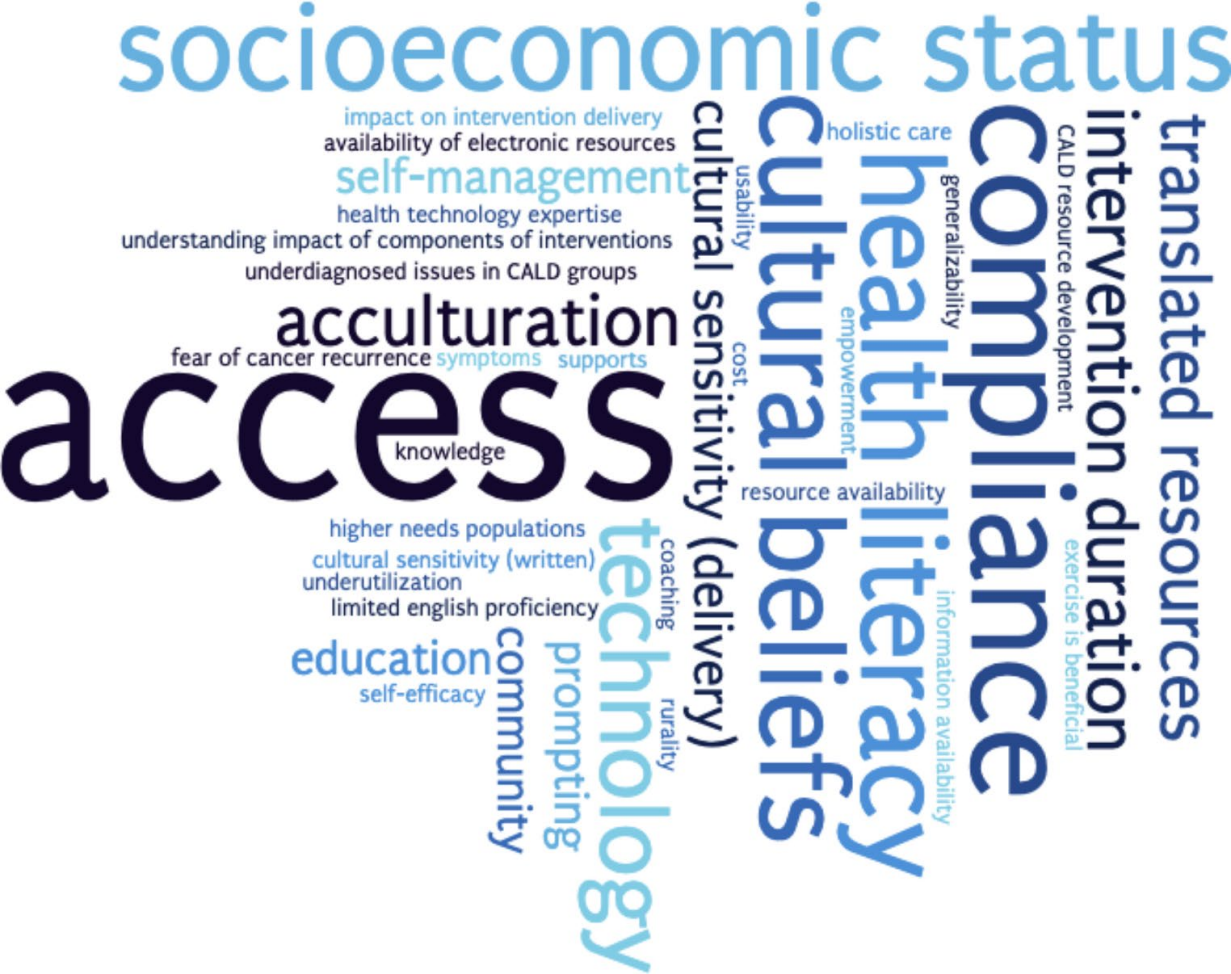
Word cloud depiction of themes arising from included studies surrounding barriers and facilitators to cancer survivorship interventions in culturally and linguistically diverse (CALD) populations

Figure 2 describes the commonly identified barriers and facilitators to survivorship care interventions from this review, which include cultural sensitivity, health literacy, socioeconomic status, acculturation, and access to care.

In more detail, cultural sensitivity as a facilitator encompassed accommodation of differing cultural beliefs such as attitudes towards cancer care and family beliefs, culturally tailored delivery of care, and availability of translated written resources and bilingual staff to convey information and training to patients. Health literacy, socioeconomic status, acculturation and access to care were noted as interrelated, participant-related barriers, and were linked to issues including: intervention usability; access to technology including internet and mobile phones; increased needs for social supports especially for those with limited education backgrounds; cost to access interventions including ability to attend scheduled appointments due to family commitments or access to transport; and negative impact of low acculturation on intervention compliance and quality of life. In particular, access to care as a barrier affected participants as well as clinicians, including: lack of available electronic resources for specific population groups; limited access to care for stigmatized conditions or marginalized populations; limited technological expertise amongst healthcare professionals and institutions; in-person delivery of interventions leading to lower compliance rates; and the effect of cancer survivor empowerment and access to health professional support in improving self-efficacy and self-management.

Other themes pertaining to barriers to care which were identified less commonly but were tied into the above included compliance, duration of the intervention, health technology expertise, required prompting and self-management, underutilization of available resources and programmes, and limited English proficiency.

## Discussion

This scoping review is the first to chart and synthesize existing literature around cancer survivorship programs or interventions available for survivors of CALD backgrounds. We found that cancer survivorship programs and interventions were associated with positive outcomes in populations of CALD backgrounds, and that one of the most crucial barriers and facilitators to delivering study interventions in this context pertains to cultural sensitivity.

Overall, there have been a variety of interventions studied in this space to date with several randomized studies demonstrating positive outcomes when adapting for patients from non-primary cultural or linguistic groups. Unfortunately, studies have generally been restricted to certain patient populations, geographic locations, and languages, namely breast cancer survivors in the United States of Spanish-speaking or specific Asian-language speaking backgrounds. These specifications make it difficult to generalise findings across different populations of cancer survivors.

Prior work across survivors of CALD backgrounds has demonstrated differences in the types and frequencies of unmet needs and challenges faced as compared with corresponding primary ethnocultural groups. Levesque et al. and Wu et al. all demonstrated upon literature review that the supportive needs of Asian patients, with a particular focus on Chinese patients, differ from their Caucasian counterparts as requiring increased informational and health system supports.^39, 40^ This was particularly associated with increased psychological needs with higher prevalence of depression and anxiety, and lower quality of life amongst Chinese patients.^40^ Similarly, increased supportive care needs in cancer survivorship have been demonstrated across non-native patients of Chinese, Hispanic, Arabic, Greek and Vietnamese descent from questionnaire-based or qualitative studies, suggesting that there are challenges faced by non-native populations across the board.^41–45^ In our review, one of the most prominent facilitators of effective care was the use of a culturally sensitive approach, thus it remains paramount that the unique needs of each cultural or linguistic group are acknowledged and health professionals be mindful not to overgeneralize findings between population groups regardless of geographic, cultural or linguistic similarities. Future work should not only focus on more prevalent non-native CALD populations, but also consider tailoring interventions towards new and emerging CALD groups.

Interventions outlined in this study were quite varied in their development, composition, and delivery. Several studies, particularly those with more complex interventional designs had a steering committee including community participants. A document analysis by Chauhan et al. revealed that out of 11 published consumer engagement frameworks in Australia between 2007 and 2019, only 4 contained sections explicitly discussing inclusion of consumers of CALD backgrounds in detail, including opportunities to improve engagement beyond language differences.^46^ Similar gaps in migrant healthcare policy addressing barriers to care were demonstrable in European populations,^47^ and although this has improved gradually over the years there remains significant heterogeneity between countries and ethnocultural populations.^48, 49^ Various studies have demonstrated that community-based participatory research methods, defined as “a partnership approach to research that equitably involves community members, organizational representatives, and researchers in all aspects of the research process”,^50^ are an effective approach to rapid development and conduct of high-quality, implementation-based research whilst simultaneously strengthening connections between academic and community members. This is especially true in conducting cross-cultural research, where it may be that researchers do not have specific insights into, nor do they have lived experiences of the issues being studied.

In our review, almost all (96%) studies had bilingual delivery (through written resources and/or bilingual speakers) of interventions available, and over half (54%) explicitly discussed staff training. As evidenced by the facilitators identified above, the cultural sensitivity of study conduct and written resources was undoubtedly an invaluable contributor to the success of the studied survivorship programs. Similarly, lower acculturation, which was only specifically studied in three studies, is thought to affect the engagement and compliance of CALD populations substantially. Whilst congruence between study populations and language is paramount to any intervention within a CALD group, the cultural aspects behind working with these populations cannot be ignored, such as differences in values, religious beliefs, health practices and socioeconomic status. This is clear from studies of professional interpreter use in the context of oncology and palliative care, which demonstrated the importance of cultural nuances alongside accurate language, however interpreters were reportedly often inadequately trained for the gravity or stress of prognostic discussions.^51–53^ In a systematic review of 10 studies of interpreter use in CALD populations within a palliative care setting, Silva et al. demonstrated that regular use of professional interpreters and bilingual staff members were associated with positive symptom and distress outcomes.^52^ Whilst supply of bilingual staff members in healthcare institutions is challenging, this review supports the existing literature that they can play a valuable role in strengthening communications, engaging communities and improving outcomes in CALD populations within the context of cancer survivorship. Given that 21 of 26 studies (81%) in this review employed relatively complex multi-modal interventions, ensuring adequate cultural and linguistic competence for all staff involved is paramount when implementing such models to maximise uptake, feasibility and optimise outcomes for these patients.

In 2019, Nekhluydov et al. developed a Quality of Cancer Survivorship Care Framework based on existing literature and previous guidelines, with the goal of informing cancer survivorship care in clinical, research and policymaking settings.^54^ The framework encompasses five domains relevant to the needs of cancer survivors, which are: prevention and surveillance for recurrences and new cancers; surveillance and management of physical effects; surveillance and management of psychosocial effects; surveillance and management of chronic medical conditions; and health promotion and disease prevention.

Based upon this framework, our review found that domains of interventions covered were heavily skewed towards surveillance and management of psychosocial (85%) and physical (77%) effects from cancer and treatment, when grouped by Cancer Survivorship care quality framework domains.^54^ Through the information provided in the available manuscripts and supplemental materials, the authors were unable to discern whether prevention and surveillance for new or recurrent cancers was a significant focus within any of the interventions. Whilst this is not ideal, particularly as concerns about non-adherence to recommended follow-up guidelines for various psychosocial, cultural or financial reasons, the authors understand that this presents a limitation within the review methodology. The remaining two interventional domains, health promotion and disease prevention (12%), and surveillance and management of chronic conditions (4%), were less well represented in the literature. The reasoning behind the emphasis on psychosocial and physical intervention content domains across studies is likely secondary to additional cultural complexities and linguistic limitations in resource dissemination and provision of support that is inherent in caring for CALD populations. Nevertheless, the uneven distributions across the different quality framework domains suggest that there is still work to be done in optimising all aspects of Cancer Survivorship care delivery in CALD populations, and this should be inclusive of areas where intervention delivery may be more challenging.

Outcome measures between studies were heterogeneous, but most used validated PROMs as either primary (65%) or secondary (15%) outcomes. Furthermore, it was encouraging to see that the majority of studies (77%) met their primary endpoints. These findings are unsurprising, as it is well-known that PROMs are often superior to physician assessments in capturing patient symptoms, particularly in the survivorship phase where symptom management and quality of life are priorities.^55, 56^ However, clinicians and researchers are now faced with a vast range of validated PROMs to choose from, each with its own pros and cons. Work is currently underway in an attempt to harmonise and protocolise PROM adoption into cancer survivorship research;^57^ however, within CALD populations there is further complexity in availability of translated PROMs and subsequent validation for clinical and academic use.^58^ As more research is conducted in various CALD communities globally, there will likely be a consequent increase in the availability of validated, translated PROMs relevant to the cancer survivorship space.

Barriers and facilitators to cancer survivorship care are an ongoing area of research worldwide. A recently published modified Delphi study outlined the highest ranked research priorities for cancer survivorship in Australia, which aligned with existing international frameworks and unmet survivorship needs.^16^ Specifically focusing on the Population Group research domains, ‘racially and ethnically diverse populations’ was ranked within the top 5 priorities for less than 30% of panellists, which likely reflects limitations in Delphi methodology and an urgent need for focused research across other priority population groups based upon prevalence.^16^ Noted priorities from this paper were also consistent with barriers and facilitators to care that were identified in our CALD-specific review, such as health services (communication, self-management, patient navigation), psychosocial (financial, distress and mood disorders), and physiological (physical activity) domains. However, our review brings to the forefront the current state of research focused on CALD populations in cancer survivorship, and there are clear unmet needs in cancer survivorship care and research in CALD populations such as fear of cancer recurrence, cognitive function, management of comorbidities and quality of survivorship care models, none of which were identified as primary subject matter within our review. The challenges moving forward for researchers in this field will be to develop high-quality, easily implementable models of survivorship care across these various domains whilst simultaneously recognising and incorporating the unique care needs of individual CALD population groups.^59^

There were several limitations to this study. Firstly, evaluation and comparison of the content, feasibility and efficacy of survivorship interventions between studies was superficial given this was a study-level analysis and there is substantial heterogeneity in population demographics, study designs, intervention delivery and endpoints. In particular, examination of specific intervention program content was not feasible and thus limited our appraisal of the depth of coverage in this area. This is to be expected given that this was a scoping review aiming to explore the available data, and specific comparisons between studies would be more appropriate in a future systematic review, perhaps focused upon Spanish-speaking immigrant breast cancer survivors where there is more literature available. Similarly, whilst it was recognised across various studies that substantial proportions of study participants were of lower socioeconomic backgrounds, due to the differences in reporting between studies this was not able to be discerned in further detail as individual patient data were not available. The intertwining of low acculturation, socioeconomic status and poor health literacy should remain at the forefront of researchers’ minds for the future. Finally, as the study search was limited to focusing on adult-onset, solid tumour cancer survivors who had completed primary curative-intent treatment, results from this work cannot be directly generalised to survivorship programs or interventions focused on paediatric-onset malignancy, haematologic cancers, or survivors with advanced/metastatic cancers; additional studies should be conducted specific to these groups.

## Conclusion

There is a growing body of evidence to support the importance of caring for cancer survivors, particularly as prognoses improve with therapeutic advances in cancer research. Globally, efforts have been made to focus research efforts on CALD populations within the context of cancer survivorship, however this review highlights that the existing literature is strongly focused on certain population demographics, with limited patient data outside the United States or breast cancer-affected populations. Nevertheless, despite the complex, multi-modal interventional designs and heterogeneity of delivery methods it is promising to see the successes that academically driven cancer survivorship units are achieving in implementing CALD-specific survivorship research. Vast gaps in knowledge and understanding of optimal survivorship care in CALD populations continue to exist, and future research should be employing culturally sensitive methods to study conduct and delivery, utilising a consumer-involved approach to maximise feasibility and uptake for novel interventions. We challenge the existing mindset that cultural and linguistic diversities pose barriers to care in patients affected by cancer, and instead posit that a more inclusive approach would be to view culture and language as unmet needs yet to be addressed by the cancer research community.

## Electronic supplementary material

Below is the link to the electronic supplementary material.

Supplementary Material 1

Supplementary Material 2

## Data Availability

The datasets generated and analysed during the current study are available from the corresponding author upon reasonable request.
