# Supplementary material for: Cancer survivorship programs for patients from culturally and linguistically diverse (CALD) backgrounds: a scoping review
Source: J Cancer Surviv. 2023 Aug 12;18(6):2052–77. doi: 10.1007/s11764-023-01442-w (PMC11502556; doi:10.1007/s11764-023-01442-w)
Supplement: Supplementary file 1 — Supplementary Material 1 [file 11764_2023_1442_MOESM1_ESM.docx]

**Supplementary Appendix – MEDLINE Search Strategy**

| 1. | Cultural Diversity/ |
| --- | --- |
| 2. | ((cultur* or linguistic*) adj4 divers*).mp. [mp=title, abstract, original title, name of substance word, subject heading word, floating sub-heading word, keyword heading word, organism supplementary concept word, protocol supplementary concept word, rare disease supplementary concept word, unique identifier, synonyms] |
| 3. | "Transients and Migrants"/ or migrant*.mp. |
| 4. | "Emigrants and Immigrants"/ or immigrant*.mp. |
| 5. | CALD.mp. |
| 6. | Limited English Proficiency/ |
| 7. | (english adj3 proficien*).mp. |
| 8. | (non english speak* or nonenglish speak*).mp. |
| 9. | (english as a second language or english as second language).mp. |
| 10. | 1 or 2 or 3 or 4 or 5 or 6 or 7 or 8 or 9 |
| 11. | Cancer Survivors/ |
| 12. | (cancer* adj3 survivor*).mp. |
| 13. | (survivor* adj3 program*).mp. |
| 14. | 11 or 12 or 13 |
| 15. | 10 and 14 |
