# Supplementary material for: Cancer survivorship programs for patients from culturally and linguistically diverse (CALD) backgrounds: a scoping review
Source: J Cancer Surviv. 2023 Aug 12;18(6):2052–77. doi: 10.1007/s11764-023-01442-w (PMC11502556; doi:10.1007/s11764-023-01442-w)
Supplement: Supplementary file 2 — Supplementary Material 2 [file 11764_2023_1442_MOESM2_ESM.docx]

**Supplementary Appendix – Reference List of Studies**

1. 2008 SNRS abstracts -- I - K. Southern Online Journal of Nursing Research. 2008;8(4):7-.

2. 2008 SNRS abstracts -- S. Southern Online Journal of Nursing Research. 2008;8(4):12-.

3. 40th Congress of the International Society of Paediatric Oncology (SIOP), Berlin, Germany, October, 2008. European Journal of Oncology Nursing. 2009;13(1):60-3.

4. Are psychosocial interventions targeting older african american cancer survivors culturally appropriate? A review of the literature. Cancer Nursing. 2012;35(2):E12-E.

5. Bibliography. Progress in Palliative Care. 2014;22(4):219-51.

6. Abbott DE, Voils CL, Fisher DA, Greenberg CC, Safdar N. Socioeconomic disparities, financial toxicity, and opportunities for enhanced system efficiencies for patients with cancer. Journal of Surgical Oncology. 2017;115(3):250-6.

7. Adams I, Huang E, Gomez SL, Allen L, Wang JHY. Traditional chinese medicine and survivorship care among chinese american breast cancer survivors. Psycho-Oncology. 2012;1):10.

8. Adams J, Rogers LQ, Demark-Wahnefried W, Schoenberger YM, Pekmezi D, Martin M, et al. Stakeholder perspectives on increasing acceptability of a web-based healthy lifestyle intervention for older cancer survivors. Journal of General Internal Medicine. 2020;35(SUPPL 1):S277.

9. Adams SA, Hebert JR, Bolick-Aldrich S, Daguise VG, Mosley CM, Modayil MV, et al. Breast cancer disparities in South Carolina: early detection, special programs, and descriptive epidemiology. Journal of the South Carolina Medical Association (1975). 2006;102(7):231-9.

10. Advani P. Disparities in adherence to breast cancer care. Dissertation Abstracts International: Section B: The Sciences and Engineering. 2017;77(12-B(E)):No Pagination Specified.

11. Advani P, Bondy M, Thompson PA, Martínez ME, Nodora JN, Vernon SW, et al. Impact of acculturation on breast cancer treatment and survivorship care among Mexican American patients in Texas. J Cancer Surviv. 2018;12(5):659-68.

12. Aitaoto N, Braun KL, Dang KL, So'a T. Cultural considerations in developing church-based programs to reduce cancer health disparities among Samoans. Ethnicity & Health. 2007;12(4):381-400.

13. Akinsulure-Smith AM. Responding to the trauma of sexual violence in asylum seekers: A clinical case study. Clinical Case Studies. 2012;11(4):285-98.

14. Alananzeh I. "cancer turned my life upside down." quality of life in Arab cancer survivors'. Supportive Care in Cancer. 2018;26(2 Supplement 1):S255.

15. Alananzeh I, Levsque J, Kwok C, Everett B. The unmet supportive care needs of cancer survivors. Supportive Care in Cancer. 2017;25(2 Supplement 1):S208.

16. Alananzeh I, Ramjan L, Kwok C, Levesque JV, Everett B. Arab-migrant Cancer Survivors' Experiences of Using Health-care Interpreters: A Qualitative Study. Asia-Pac. 2018;5(4):399-407.

17. Alananzeh IM, Levesque JV, Kwok C, Salamonson Y, Everett B. The unmet supportive care needs of arab Australian and arab jordanian cancer survivors: An international comparative survey. Cancer Nursing. 2019;42(3):E51-E60.

18. Aldridge L, McGrane J, Butow P, Girgis A, Jefford M, Schofield P, et al. Unmet needs in Chinese (Mandarin and Cantonese), Greek, and Arabic-speaking cancer survivors in Australia. Psycho-Oncology. 2010;2):S4-S5.

19. Aldridge LJ, Butow P, Bell M, McGrane J, Jefford M, Schofield P, et al. The case for culturally competent navigators: Examining health disparities, distress and unmet need in Chinese (Mandarin and Cantonese), Greek, and Arabic speaking cancer survivors in Australial. Asia-Pacific Journal of Clinical Oncology. 2010;3):236.

20. Alexander J, Kwon HT, Strecher R, Bartholomew J. Multicultural media outreach: increasing cancer information coverage in minority communities. Journal of Cancer Education. 2013;28(4):744-7.

21. Allen EM, Loftus JO. Barriers to recommended screening among U.S. immigrants: A literature review. Cancer Epidemiology Biomarkers and Prevention Conference: 8th AACR Conference on the Science of Health Disparities in Racial/Ethnic Minorities and the Medically Underserved Atlanta, GA United States. 2016;25(3 Supplement).

22. Almegewly W, Gould D, Anstey S. Hidden voices: an interpretative phenomenological analysis of the experience of surviving breast cancer in Saudi Arabia. Journal of Research in Nursing. 2019;24(1/2):122-32.

23. Amari SPF, Bell K, Walker BP, Vaughn YV, Faison D, Smith JA. Preparing the future workforce for comprehensive cancer control. Journal of Women's Health. 2013;22(3):47.

24. Andriessen K. Survivors After Suicide: A comprehensive suicide survivor program in Flanders, Belgium. Grief after suicide: Understanding the consequences and caring for the survivors. 2011:495-504.

25. Anonymous. This journal issue contains abstracts that have been accepted for presentation as the 8th Annual Conference of the International Society for Quality of Life Research (ISOQOL). Quality of Life Research. 2001;10(3):193-306.

26. Anonymous. Flourishing or flailing? The impact of cancer on survivors and their caregivers across the cancer trajectory. Psycho-Oncology. 2010;2):S3.

27. Anonymous. AACR International Conference on the Science of Cancer Health Disparities 2011. Cancer Epidemiology Biomarkers and Prevention Conference: American Association for Cancer Research, AACR International Conference on the Science of Cancer Health Disparities. 2011;20(10 Meeting Abstracts).

28. Anonymous. AACR Virtual Conference: 14th AACR Conference on the Science of Cancer Health Disparities in Racial/Ethnic Minorities and the Medically Underserved. Cancer Epidemiology Biomarkers and Prevention Conference: 14th AACR Conference on the Science of Cancer Health Disparities in Racial/Ethnic Minorities and the Medically Underserved Virtual. 2022;31(1 SUPPL).

29. Anonymous. On the path to equity in cancer research and care. Trends in Cancer. 2022;8(4):262-5.

30. Aparicio-Ting F, Ramirez AG. Breast and cervical cancer knowledge, attitudes, and screening practices of hispanic women diagnosed with cancer. Journal of Cancer Education. 2003;18(4):230-6.

31. Arega MA, Dee EC, Muralidhar V, Nguyen PL, Franco I, Sanford NN, et al. Mental Distress and Mental Health Services Receipt in Foreign-Born Survivors of Cancer: a National Health Interview Survey Analysis. Journal of General Internal Medicine. 2021;36(8):2495-8.

32. Arega MA, Dee EC, Muralidhar V, Nguyen PL, Franco II, Sanford NN, et al. Mental Distress and Mental Health Services Receipt in Foreign Born Survivors of Cancer: A National Health Interview Survey Analysis. International Journal of Radiation Oncology Biology Physics. 2020;108(3 Supplement):e403-e4.

33. Arega MA, Dee EC, Muralidhar V, Nguyen PL, Franco II, Sanford NN, et al. Mental distress and mental health services receipt in foreign-born survivors of cancer: A national health interview survey analysis. Journal of Clinical Oncology Conference. 2020;38(15).

34. Ariyanto AA, Muluk H, Newcombe P, Piercy FP, Poerwandari EK, Suradijono SHR. Diversity in unity: Perspectives from psychology and behavioral sciences. Diversity in unity: Perspectives from psychology and behavioral sciences. 2018:xiv, 648.

35. Ashing K. A prospective study of health-related quality of life among Chinese-American breast cancer survivors. Psycho-Oncology. 2015;2):19-20.

36. Ashing K, Rosales M. A telephonic-based trial to reduce depressive symptoms among Latina breast cancer survivors. Psycho-Oncology. 2014;23(5):507-15.

37. Ashing KT, George M, Jones V. Health-related quality of life and care satisfaction outcomes: Informing psychosocial oncology care among Latina and African-American young breast cancer survivors. Psycho-Oncology. 2018;27(4):1213-20.

38. Ashing KT, Padilla G, Tejero J, Kagawa-Singer M. Understanding the breast cancer experience of Asian American women. Psycho-Oncology. 2003;12(1):38-58.

39. Ashing-Giwa K. Examining demographic and socio-ecological influences on depressive outcomes among latina American Breast Cancer survivors. Psycho-Oncology. 2011;2):263-4.

40. Ashing-Giwa K, Lim J, Young L, Yeung S. Examining health-related quality of life and access to health care outcomes among Chinese-, Korean-, and Filipino-American breast cancer survivors. Psycho-Oncology. 2010;1):S61.

41. Ashing-Giwa K, Lim JW, Young L, Yeung S. Examining health-related quality of life and access to health care outcomes among chinese, Korean, and filipino-American breast cancer survivors. Psycho-Oncology. 2011;2):262-3.

42. Ashing-Giwa K, Rosales M. A cross-cultural validation of patient-reported outcomes measures: a study of breast cancers survivors. Quality of life research : an international journal of quality of life aspects of treatment, care and rehabilitation. 2013;22(2):295-308.

43. Ashing-Giwa K, Rosales M. Evaluation of therapeutic care delay among Latina- and European-American cervical cancer survivors. Gynecologic Oncology. 2013;128(2):160-5.

44. Ashing-Giwa KT. Can a culturally responsive model for research design bring us closer to addressing participation disparities? Lessons learned from cancer survivorship studies. Ethn Dis. 2005;15(1):130-7.

45. Ashing-Giwa KT. Enhancing physical well-being and overall quality of life among underserved Latina-American cervical cancer survivors: Feasibility study. Journal of Cancer Survivorship. 2008;2(3):215-23.

46. Ashing-Giwa KT, Kim J, Tejero JS. Measuring quality of life among cervical cancer survivors: Preliminary assessment of instrumentation validity in a cross-cultural study. Quality of Life Research. 2008;17(1):147-57.

47. Ashing-Giwa KT, Padilla G, Tejero J, Kraemer J, Wright K, Coscarelli A, et al. Understanding the breast cancer experience of women: A qualitative study of African American, Asian American, Latina and Caucasian cancer survivors. Psycho-Oncology. 2004;13(6):408-28.

48. Ashing-Giwa KT, Padilla GV, Tejero JS, Kim J. Breast cancer survivorship in a multiethnic sample: challenges in recruitment and measurement. Cancer. 2004;101(3):450-65.

49. Aung LL, Sabai SM, Yeoh AEJ, Chan MY, Quah TC. An update from singapore childhood cancer survivor study (Singapore-ccss). Pediatric Blood and Cancer. 2012;59(6):1110.

50. Aung LL, Saw MS, Chan MY, Yeoh AEJ, Tan AM, Quah TC. An update from Singapore childhood cancer survivor study (Singapore-CCSS). Proceedings of Singapore Healthcare. 2012;1):S283.

51. Axelrod D. The psychological and psychiatric implications during and after a diagnosis of cancer: Advocating on their behalf. Primary Psychiatry. 2008;15(10):35-7.

52. Aycinena AC, Jennings KA, Gaffney AO, Koch PA, Contento IR, Gonzalez M, et al. ¡Cocinar Para Su Salud! Development of a Culturally Based Nutrition Education Curriculum for Hispanic Breast Cancer Survivors Using a Theory-Driven Procedural Model. Health Educ Behav. 2017;44(1):13-22.

53. Aziz N, Rahman A. Racial and ethnic disparities in patient-provider communication and perceived care quality among long-term cancer survivors. Psycho-Oncology. 2018;27(Supplement 2):21-2.

54. Bach SC, Goddard B, Nathanson A, Ades V. Perceptions of female genital cutting among a population of female survivors of torture. International Journal of Gynecology and Obstetrics. 2018;143(Supplement 3):871-2.

55. Bache RA, Bhui KS, Dein S, Korszun A. African and Black Caribbean origin cancer survivors: A qualitative study of the narratives of causes, coping and care experiences. Ethnicity and Health. 2012;17(1-2):187-201.

56. Baik SH, Oswald LB, Buscemi J, Buitrago D, Iacobelli F, Perez-Tamayo A, et al. Patterns of Use of Smartphone-Based Interventions Among Latina Breast Cancer Survivors: Secondary Analysis of a Pilot Randomized Controlled Trial. JMIR Cancer. 2020;6(2):e17538.

57. Bailey S, Lin J. The association of osteoporosis knowledge and beliefs with preventive behaviors in postmenopausal breast cancer survivors. BMC Women's Health. 2021;21(1) (no pagination).

58. Bal DG. Cancer and social justice: A demographic, economic, historic, sociocultural, and ethical perspective. Cancer. 2005;104(12 SUPPL.):2891-4.

59. Bamidele OO, McCaughan E. A constructivist grounded theory study on decision‐making for treatment choice among Black African and Black Caribbean prostate cancer survivors. European Journal of Cancer Care. 2022;31(1):1-9.

60. Banerjee SC, Camacho-Rivera M, Haque N, Flynn L, Thomas J, Smith P, et al. Understanding cognitive and emotional illness representations of South Asian head and neck cancer survivors: a qualitative study. Ethnicity & health. 2022;27(1):119-36.

61. Baron-Epel O, Friedman N, Lernau O. Fatalism and Mammography in a Multicultural Population. Oncology Nursing Forum. 2009;36(3):353-61.

62. Barrett M, Uí Dhuibhir P, Njoroge C, Wickham S, Buchanan P, Aktas A, et al. Diet and nutrition information on nine national cancer organisation websites: A critical review. European Journal of Cancer Care. 2020;29(5).

63. Bartley N, Davies G, Butow P, Napier CE, Schlub T, Ballinger ML, et al. Fear of cancer recurrence in patients undergoing germline genome sequencing. Supportive Care in Cancer. 2021;29(12):7289-97.

64. Bava L, Freyer DR, Radbill LM, Johns AL. Association of language proficiency, sociodemographics, and neurocognitive functioning in dual-language Latino survivors of childhood acute lymphoblastic leukemia and lymphoma. Pediatric Blood and Cancer. 2021;68(9) (no pagination).

65. Bava L, Johns A, Freyer DR, Ruccione K. Development of a Culturally Competent Service to Improve Academic Functioning for Latino Survivors of Acute Lymphoblastic Leukemia: Methodological Considerations. Journal of pediatric oncology nursing : official journal of the Association of Pediatric Oncology Nurses. 2017;34(3):222-9.

66. Bava L, Johns A, Kayser K, Freyer DR. Cognitive outcomes among Latino survivors of childhood acute lymphoblastic leukemia and lymphoma: A cross-sectional cohort study using culturally competent, performance-based assessment. Pediatric Blood and Cancer. 2018;65(2) (no pagination).

67. Beagan BL, Chapman GE. Family influences on food choice: Context of surviving breast cancer. Journal of Nutrition Education and Behavior. 2004;36(6):320-6.

68. Bell ML, Butow PN, Goldstein D. Informatively missing quality of life and unmet needs sex data for immigrant and Anglo-Australian cancer patients and survivors. Quality of Life Research. 2013;22(10):2757-60.

69. Belle F, Wengenroth L, Weiss A, Sommer G, Ansari M, Bochud M, et al. Low adherence to dietary recommendations in adult childhood cancer survivors. Clinical Nutrition. 2017;36(5):1266-74.

70. Belle FN, Weiss A, Schindler M, Goutaki M, Bochud M, Zimmermann K, et al. Overweight in childhood cancer survivors: The Swiss childhood cancer survivor study. American Journal of Clinical Nutrition. 2018;107(1):3-11.

71. Ben David R, Biderman A, Sherf M, Zamstein O, Dreiher J. Elevated cancer risk in Holocaust survivors residing in Israel: A retrospective cohort study. European Journal of Cancer. 2018;95:85-92.

72. Bender JL, Wiljer D, Sawka A, Alkazaz N, Brierley JD. Thyroid cancer survivors' supportive care needs: A cross-sectional survey. Thyroid. 2012;1):A71.

73. Bhardwaj T, Koffman J. Non-pharmacological interventions for management of fatigue among children with cancer: Systematic review of existing practices and their effectiveness. BMJ Supportive and Palliative Care. 2017;7(4):404-14.

74. Blinder V, Murphy M, Vahdat L, Gold H, de Melo-Martin I, Hayes M, et al. Employment after a breast cancer diagnosis: A qualitative study of ethnically diverse urban women. Journal of Community Health: The Publication for Health Promotion and Disease Prevention. 2012;37(4):763-72.

75. Blinder VS, Eberle CE, Patil S, Ramirez J, McNish T, Min SH, et al. Ethnic disparities in the impact of breast cancer on the workforce: A pilot study. Journal of Clinical Oncology Conference. 2015;33(15 SUPPL. 1).

76. Blinder VS, Eberle CE, Tran C, Bao T, Malik M, Jung G, et al. Use of patient-reported controls for secular trends to study disparities in cancer-related job loss. Journal of cancer survivorship : research and practice. 2021;15(5):685-95.

77. Blinder VS, Murphy MM, Vahdat LT, Gold HT, de Melo-Martin I, Hayes MK, et al. Employment after a breast cancer diagnosis: a qualitative study of ethnically diverse urban women. Journal of community health. 2012;37(4):763-72.

78. Blinder VS, Patil S, Thind A, Diamant A, Hudis CA, Basch E, et al. Return to work in low-income Latina and non-Latina white breast cancer survivors: A 3-year longitudinal study. Cancer. 2012;118(6):1664-74.

79. Block R, Frohnmayer A, Cervantes O, Dahlstrom M, Hayes-Lattin B. Experiences of cancer and depression among adolescent and young adult latino men. Psycho-Oncology. 2012;1):74.

80. Bonilla J, Escalera C, Santoyo-Olsson J, Samayoa C, Ortiz C, Stewart AL, et al. The importance of patient engagement to quality of breast cancer care and health-related quality of life: a cross-sectional study among Latina breast cancer survivors in rural and urban communities. BMC Women's Health. 2021;21(1) (no pagination).

81. Boyd T. Fair care. Nursecom Nursing Spectrum (New York/New Jersey Metro). 2011;23(15):16-8.

82. Bright CJ, Reulen RC, Winter DL, Stark DP, McCabe MG, Edgar AB, et al. Risk of subsequent primary neoplasms in survivors of adolescent and young adult cancer (Teenage and Young Adult Cancer Survivor Study): a population-based, cohort study. The Lancet Oncology. 2019;20(4):531-45.

83. Brotto LA, Kingsberg SA. Sexual consequences of cancer survivorship. Handbook of clinical sexuality for mental health professionals , 2nd ed. 2010:329-47.

84. Buki L, Reich M, Lehardy E. Latina breast cancer survivors' psychosocial determinants of body image acceptance. Psycho-Oncology. 2016;2):74-5.

85. Buki LP, Garces D, Hinestrosa M, Kogan L, Carrillo IY, French B. Latina breast cancer survivors' lived experiences: Diagnosis, treatment, and beyond. Cultural Diversity and Ethnic Minority Psychology. 2008;14(2):163-7.

86. Buki LP, Garces DM, Hinestrosa MC, Kogan L, Carrillo IY, French B. Latina Breast Cancer Survivors' Lived Experiences: Diagnosis, Treatment, and Beyond. Cultural Diversity and Ethnic Minority Psychology. 2008;14(2):163-7.

87. Buki LP, Piedra LM. Creating infrastructures for Latino mental health. Creating infrastructures for Latino mental health. 2011:xxviii, 276.

88. Buki LP, Rivera-Ramos ZA, Kanagui-Munoz M, Heppner PP, Ojeda L, Lehardy EN, et al. "I never heard anything about it": Knowledge and psychosocial needs of Latina breast cancer survivors with lymphedema. Women's Health. 2021;17(no pagination).

89. Buki LP, Schwartz JR, McInerney EEW. Creating community: Expanding access to psychosocial programs for Latina breast cancer survivors. Psycho Oncology. 2021.

90. Burhansstipanov L, Burhansstipanov L. Community-driven Native American cancer survivors' quality of life research priorities. Journal of Cancer Education. 2005;20:7-11.

91. Burke NJ, Napoles TM, Banks PJ, Orenstein FS, Luce JA, Joseph G. Survivorship care plan information needs: Perspectives of safety-net breast cancer patients. PLoS ONE. 2016;11(12) (no pagination).

92. Burke NJ, Villero O, Guerra C. Passing through: meanings of survivorship and support among Filipinas with breast cancer. Qualitative health research. 2012;22(2):189-98.

93. Buscemi J, Buitrago D, Iacobelli F, Penedo F, Maciel C, Guitleman J, et al. Feasibility of a Smartphone-based pilot intervention for Hispanic breast cancer survivors: a brief report. Translational Behavioral Medicine. 2019;9(4):638-45.

94. Butow P, Sze M, Goldstein D, Jefford M. Having cancer in a country not your own: The Chinese immigrant story in Australia. Psycho-Oncology. 2015;2):20.

95. Butow PN, Aldridge L, Bell M, Sze M, Eisenbruch M, King M, et al. Cancer survivorship outcomes in immigrants. Journal of Clinical Oncology Conference. 2012;30(15 SUPPL. 1).

96. Butow PN, Aldridge L, Bell ML, Sze M, Eisenbruch M, Jefford M, et al. Inferior health-related quality of life and psychological well-being in immigrant cancer survivors: A population-based study. European Journal of Cancer. 2013;49(8):1948-56.

97. Caicedo L. Coordinating navigation across health care systems: Site specific case from Nueva Vida, Inc. Psycho-Oncology. 2013;2):141-2.

98. Campesino M. Exploring perceptions of cancer care delivery among older Mexican American adults. Oncology nursing forum. 2009;36(4):413-20.

99. Campesino M, Koithan M, Ruiz E, Glover JU, Juarez G, Choi M, et al. Surgical treatment differences among latina and African American breast cancer survivors. Oncology Nursing Forum. 2012;39(4):E324-E31.

100. Campesino M, Ruiz E, Glover J, Saenz D, Larkey L, Krouse R, et al. Perceptions of discrimination in cancer care delivery among Latina and African American breast cancer survivors. Psycho-Oncology. 2010;1):S12.

101. Campesino M, Ruiz E, Glover JU, Koithan M. Counternarratives of Mexican-origin women with breast Cancer. Advances in Nursing Science. 2009;32(2):E57-E67.

102. Cao W, Cho H. Culture in cancer survivorship interventions for Asian Americans: A systematic review and critical analyses. Asian American Journal of Psychology. 2021;12(1):65-75.

103. Carr L. Treating diverse communities. Journal of Thoracic Oncology. 2015;2):S76.

104. Casaubon JT, Kuehn RB, Pesek SE, Raker CA, Edmonson DA, Stuckey A, et al. Breast-Specific Sensuality and Appearance Satisfaction: Comparison of Breast-Conserving Surgery and Nipple-Sparing Mastectomy. Journal of the American College of Surgeons. 2020;230(6):990-8.

105. Casillas J, Kahn KL, Doose M, Landier W, Bhatia S, Hernandez J, et al. Transitioning childhood cancer survivors to adult-centered healthcare: insights from parents, adolescent, and young adult survivors. Psycho-Oncology. 2010;19(9):982-90.

106. Chan A, Gan YX, Oh SK, Ng T, Shwe M, Chan R, et al. A culturally adapted survivorship programme for Asian early stage breast cancer patients in Singapore: A randomized, controlled trial. Psycho-Oncology. 2017;26(10):1654-9.

107. Chang A, Scherb H, Causevic S. Analysis of refugee mental health screening and referral processes at the newcomers health program, San Francisco general hospital's refugee medical clinic: A quality improvement study. Annals of Global Health. 2016;82(3):409.

108. Chapman GE, Beagan B. Women's perspectives on nutrition, health, and breast cancer. Journal of Nutrition Education and Behavior. 2003;35(3):135-41.

109. Chawla N. South Asian women with breast cancer: Navigating cancer care and the role of social capital in obtaining cancer resources. Dissertation Abstracts International: Section B: The Sciences and Engineering. 2012;73(3-B):1512.

110. Chee W, Lee Y, Im EO, Chee E, Tsai HM, Nishigaki M, et al. A culturally tailored Internet cancer support group for Asian American breast cancer survivors: A randomized controlled pilot intervention study. J Telemed Telecare. 2017;23(6):618-26.

111. Chee W, Lee Y, Ji X, Chee E, Im EO. The Preliminary Efficacy of a Technology-Based Cancer Pain Management Program Among Asian American Breast Cancer Survivors. Comput Inform Nurs. 2020;38(3):139-47.

112. Chen A, Huang X, Leng J, Gany F, Lui F, Breitbart W. Adapting Meaning-Centered Psychotherapy in Advanced Cancer for the Chinese Immigrant Population. Journal of Immigrant & Minority Health. 2018;20(3):680-6.

113. Chou FY, Kuang LY, Lee J, Yoo GJ, Fung LC. Challenges in Cancer Self-management of Patients with Limited English Proficiency. Asia-Pac. 2016;3(3):259-65.

114. Christ G, Messner C, Behar L. Handbook of oncology social work: Psychosocial care for people with cancer. Handbook of oncology social work: Psychosocial care for people with cancer. 2015:xxxi, 837.

115. Chu Q, Wong CCY, Chen L, Shin LJ, Chen L, Lu Q. Self-stigma and quality of life among Chinese American breast cancer survivors: A serial multiple mediation model. Psychooncology. 2021;30(3):392-9.

116. Chu Q, Wong CCY, Lu Q. Acculturation Moderates the Effects of Expressive Writing on Post-Traumatic Stress Symptoms Among Chinese American Breast Cancer Survivors. International journal of behavioral medicine. 2019;26(2):185-94.

117. Chung J, Kulkarni GS, Bender J, Breau RH, Guttman D, Maganti M, et al. Modifiable lifestyle behaviours impact the health-related quality of life of bladder cancer survivors. BJU International. 2020;125(6):836-42.

118. Chung J, Kulkarni GS, Morash R, Matthew A, Papadakos J, Breau RH, et al. Assessment of quality of life, information, and supportive care needs in patients with muscle and non-muscle invasive bladder cancer across the illness trajectory. Supportive Care in Cancer. 2019;27(10):3877-85.

119. Chung LK, Cimprich B, Janz NK, Mills-Wisneski SM. Breast cancer survivorship program: Testing for cross-cultural relevance. Cancer Nursing. 2009;32(3):236-45.

120. Coenen P, Zegers AD, Stapelfeldt CM, Maaker‐Berkhof M, Abma F, Beek AJ, et al. Cross‐cultural translation and adaptation of the Readiness for Return To Work questionnaire for Dutch cancer survivors. European Journal of Cancer Care. 2021;30(2):1-12.

121. Cohen MZ, Rozmus CL, Mendoza TR, Padhye NS, Neumann J, Gning I, et al. Symptoms and quality of life in diverse patients undergoing hematopoietic stem cell transplantation. Journal of Pain and Symptom Management. 2012;44(2):168-80.

122. Connor AE, Dibble KE. Disparities in perceived healthcare discrimination among BRCA1/2-positive women from disadvantaged health populations. Cancer Epidemiology Biomarkers and Prevention Conference: 14th AACR Conference on the Science of Cancer Health Disparities in Racial/Ethnic Minorities and the Medically Underserved Virtual. 2022;31(1 SUPPL).

123. Cook O, McIntyre M, Recoche K, Lee S. Experiences of gynecological cancer patients receiving care from specialist nurses: a qualitative systematic review. JBI Database System Rev Implement Rep. 2017;15(8):2087-112.

124. Cormio C, Romito F, Giotta F, Mattioli V. Post-traumatic growth in the Italian experience of long-term disease-free cancer survivors. Stress and Health: Journal of the International Society for the Investigation of Stress. 2015;31(3):189-96.

125. Corvin J, Coreil J, Nupp R, Dyer K. ETHNIC DIFFERENCES IN CULTURAL MODELS OF BREAST CANCER SUPPORT GROUPS. International Journal of Self Help & Self Care. 2013;7(2):193-215.

126. Costas-Muñiz R, Hunter-Hernández M, Garduño-Ortega O, Morales-Cruz J, Gany F. Ethnic differences in psychosocial service use among non-Latina white and Latina breast cancer survivors. Journal of Psychosocial Oncology. 2017;35(4):424-37.

127. Cragun D, Weidner A, Lewis C, Bonner D, Kim J, Vadaparampil ST, et al. Racial disparities in BRCA testing and cancer risk management across a population-based sample of young breast cancer survivors. Cancer. 2017;123(13):2497-505.

128. D'Souza MS, O'Mahony J, Karkada SN. Effectiveness and meaningfulness of breast cancer survivorship and peer support for improving the quality of life of immigrant women: A mixed methods systematic review protocol. Clinical Epidemiology and Global Health. 2021;10 (no pagination).

129. Danesi G, Bourquin C, Stiefel F, Zaman K, Saraga M. The isolation of cancer survivors. European Journal of Cancer Care Vol 29(2), 2020, ArtID e13194. 2020;29(2).

130. de la Cour CD, Dehlendorff C, von Buchwald C, Garset-Zamani M, Gronhoj C, Carlander ALF, et al. Non-aspirin NSAIDs and head and neck cancer mortality in a Danish nationwide cohort study. Cancer Epidemiology. 2022;77 (no pagination).

131. De Mendoza AH, Trillos SG, Schwartz M, Peshkin B, Hamilton HE, Fiallos K, et al. Developing a cultural adaptation of a telephone genetic counseling intervention for Latina women at-risk of hereditary breast and ovarian cancer. Journal of Clinical and Translational Science. 2018:68.

132. Deen L, Buddeke J, Vaartjes I, Bots ML, Norredam M, Agyemang C. Ethnic differences in cardiovascular morbidity and mortality among patients with breast cancer in the Netherlands: A register-based cohort study. BMJ Open. 2018;8(8) (no pagination).

133. Desai S, Vazquez M, Hernandez DC, Reitzel LR, Murillo R. Lower acculturation is associated with lower aerobic physical activity among recent Latinoimmigrant cancer survivors. Cancer Epidemiology Biomarkers and Prevention Conference: 12th AACR Conference on the Science of Cancer Health Disparities in Racial/Ethnic Minorities and the Medically Underserved San Francisco, CA United States. 2020;29(6 SUPPL 2).

134. Desautels D, Czaykowski P, Nugent Z, Demers AA, Mahmud SM, Singh H. Risk of colorectal cancer after the diagnosis of prostate cancer: A population-based study. Cancer. 2016;122(8):1254-60.

135. Dgani-Ratsaby A. The effects of cultural and familial factors on severity of trauma and treatment outcome among a multicultural population of refugee survivors of torture. Dissertation Abstracts International: Section B: The Sciences and Engineering. 2012;73(2-B):1245.

136. Dhabhar F. Stress, anxiety, and susceptibility to squamous cell carcinoma: Role of immune mediators. Psychoneuroendocrinology. 2016;71(Supplement 1):1-2.

137. Dhabhar FS. Stress, anxiety, and susceptibility to squamous cell carcinoma: Role of immune mediators. Cancer Research Conference: 103rd Annual Meeting of the American Association for Cancer Research, AACR. 2012;72(8 SUPPL. 1).

138. Diaz G, Ashing KT, Chavez NR. Understanding the association of psychological distress and other comorbidities with patient-centered outcomes in latina breast cancer survivors. Psycho-Oncology. 2016;2):125-6.

139. Dibble KE, Connor AE. Evaluation of disparities in anxiety, depression, and cancer empowerment in a study of BRCA1/2-positive females in the United States. Cancer Research Conference. 2021;82(4 SUPPL).

140. Dieli-Conwright CM, Courneya KS, Demark-Wahnefried W, Sami N, Lee K, Sweeney FC, et al. Aerobic and resistance exercise improves physical fitness, bone health, and quality of life in overweight and obese breast cancer survivors: A randomized controlled trial 11 Medical and Health Sciences 1117 Public Health and Health Services. Breast Cancer Research. 2018;20(1) (no pagination).

141. Dieli-Conwright CM, Fox FS, Tripathy D, Sami N, Van Fleet J, Buchanan TA, et al. Hispanic ethnicity as a moderator of the effects of aerobic and resistance exercise on physical fitness and quality-of-life in breast cancer survivors. J Cancer Surviv. 2021;15(1):127-39.

142. Dieli-Conwright CM, Sweeney FC, Courneya KS, Tripathy D, Sami N, Lee K, et al. Hispanic ethnicity as a moderator of the effects of aerobic and resistance exercise in survivors of breast cancer. Cancer. 2019;125(6):910-20.

143. Dixit N, Wong P, McBride R, Hernandez D, Cicerelli B. Survivorship care plans in safety net: Bridging the barriers. Journal of Clinical Oncology Conference. 2018;36(7).

144. Dohan D, Szolarova R, Walcer B. How a cancer education group serves the underserved: 'Family' ties and battling cancer. Patient Education and Counseling. 2012;87(2):212-6.

145. Eakin E, Demark-Wahnefried W. Changing health behaviors after treatment. Psycho-oncology , 3rd ed. 2015:635-43.

146. Ebunlomo EO. Understanding strategies for promoting breast health among African immigrant women. Dissertation Abstracts International: Section B: The Sciences and Engineering. 2015;76(4-B(E)):No Pagination Specified.

147. Edmonds MC, Sutton AL, Cummings Y, Sheppard VB. Opportunities to Improve Women's Health: Engaging Racial/Ethnic Diverse Women to Provide Biospecimens for Research. Journal of Women's Health (15409996). 2021;30(9):1321-7.

148. Elimimian E, Elson L, Bilani N, Farrag SE, Dwivedi AK, Pasillas R, et al. Long-Term Effect of a Nonrandomized Psychosocial Mindfulness-Based Intervention in Hispanic/Latina Breast Cancer Survivors. Integrative Cancer Therapies. 2020;19:1534735419890682.

149. Erwin DO, Spatz TS, Stotts RC, Hollenberg JA. Increasing mammography practice by African American women. Cancer Practice. 1999;7(2):78-85.

150. Eunyoung ES, Hyewon K, Jiyoung K, Hyunsun K, Kwi Ock P, Bo L, et al. Outcomes of a culturally responsive health promotion program for elderly Korean survivors of gastrointestinal cancers: A randomized controlled trial. Geriatric Nursing. 2013;34(6):445-52.

151. Farrell M. DR. VERED STEARNS BELIEVES THAT DIVERSITY IN THE WORKFORCE PROVIDES MULTIPLE PERSPECTIVES IN CONFRONTING CHALLENGES, ENHANCING BREAKTHROUGHS. ASCO Connection. 2022;13(2):20-3.

152. Farren AT. Leininger’s Ethnonursing Research Methodology and Studies of Cancer Survivors. Journal of Transcultural Nursing. 2015;26(4):418-27.

153. Fatone AM. Measurement of quality of life in minority breast cancer survivors. Dissertation Abstracts International: Section B: The Sciences and Engineering. 2002;63(1-B):521.

154. Faul LA, Adams I, Gomez S, Allen L, Wang JHY. Comparison of cancer beliefs and coping after treatment among immigrant chinese, U.S.-born chinese and caucasian breast cancer survivors. Psycho-Oncology. 2013;2):29.

155. Feigelson HS, McMullen CK, Madrid S, Sterrett AT, Powers JD, Blum-Barnett E, et al. Optimizing patient-reported outcome and risk factor reporting from cancer survivors: a randomized trial of four different survey methods among colorectal cancer survivors. Journal of cancer survivorship : research and practice. 2017;11(3):393-400.

156. Findley PA. Global considerations. Handbook of cancer survivorship. 2007:449-79.

157. Fisher W. Measuring hot flashes: Examination of an alternate criterion for ambulatory hot flash detection in post-menopausal women. Dissertation Abstracts International: Section B: The Sciences and Engineering. 2014;75(5-B(E)):No Pagination Specified.

158. Fitzpatrick TR. Treating vulnerable populations of cancer survivors: A biopsychosocial approach. (2016) Treating vulnerable populations of cancer survivors: A biopsychosocial approach xv, 160 pp Cham, Switzerland: Springer International Publishing/Springer Nature; Switzerland. 2016.

159. Fleischer A. Commentary: Hidden voices: an interpretative phenomenological analysis of the experience of surviving breast cancer in Saudi Arabia. Journal of Research in Nursing. 2019;24(1/2):133-4.

160. Galvan N, Buki LP, Garces DM. Suddenly, a carriage appears: Social support needs of latina breast cancer survivors. Journal of Psychosocial Oncology. 2009;27(3):361-82.

161. Gansler T. Internet interventions help patients newly diagnosed with cancer improve their quality of life. CA Cancer Journal for Clinicians. 2018;68(3):180-1.

162. Gany F. The Integrated Cancer Care Access Network (ICCAN) addressing social and economic barriers to receipt of optimal cancer care among underserved immigrant and other minorities in New York City. Psycho-Oncology. 2015;2):24-5.

163. Garcia-Jimenez M, Santoyo-Olsson J, Ortiz C, Lahiff M, Sokal-Gutierrez K, Napoles AM. Acculturation, inner peace, cancer self-efficacy, and self-rated health among Latina breast cancer survivors. Journal of Health Care for the Poor and Underserved. 2014;25(4):1586-602.

164. Gerges M, Smith AB, Durcinoska I, Yan H, Girgis A. Exploring levels and correlates of health literacy in Arabic and Vietnamese immigrant patients with cancer and their English-speaking counterparts in Australia: A cross-sectional study protocol. BMJ Open. 2018;8(7) (no pagination).

165. Gianinazzi ME, Rueegg CS, Vetsch J, Luer S, Kuehni CE, Michel G. Cancer's positive flip side: posttraumatic growth after childhood cancer. Supportive Care in Cancer. 2016;24(1):195-203.

166. Giardini M. Lost and found: Cross cultural perspectives on breast cancer survivor work. Dissertation Abstracts International: Section B: The Sciences and Engineering. 2013;74(4-B(E)):No Pagination Specified.

167. Gibson LM, Hendricks CS. Integrative review of spirituality in African American breast cancer survivors. Abnf J. 2006;17(2):67-72.

168. Girgis A, Delaney G, Arnold A, Miller A, Carolan M, Kaadan N, et al. The PROMPT-Care Project: Working towards systematic collection of patient reported health outcomes. Journal of Medical Imaging and Radiation Oncology. 2015;1):72.

169. Glenn BA, Hamilton AS, Nonzee NJ, Maxwell AE, Crespi CM, Ryerson AB, et al. Obesity, physical activity, and dietary behaviors in an ethnically-diverse sample of cancer survivors with early onset disease. Journal of Psychosocial Oncology. 2018;36(4):418-36.

170. Gomez SL, Clarke CA, Shema SJ, Chang ET, Keegan THM, Glaser SL. Disparities in breast cancer survival among Asian women by ethnicity and immigrant status: a population-based study. American Journal of Public Health. 2010;100(5):861-9.

171. Gonzales FA, Hurtado-de-Mendoza A, Santoyo-Olsson J, Napoles AM. Do coping strategies mediate the effects of emotional support on emotional well-being among Spanish-speaking Latina breast cancer survivors? Psycho-Oncology. 2016;25(11):1286-92.

172. Gonzalez P, Lim JW, Wang-Letzkus M, Flores KF, Allen KM, Castaneda SF, et al. Breast Cancer Cause Beliefs: Chinese, Korean, and Mexican American Breast Cancer Survivors. Western journal of nursing research. 2015;37(8):1081-99.

173. Goodman LA, Fauci JE, Sullivan CM, DiGiovanni CD, Wilson JM. Domestic violence survivors' empowerment and mental health: Exploring the role of the alliance with advocates. American Journal of Orthopsychiatry. 2016;86(3):286-96.

174. Gotay CC. Quality of life research in Hawaii's cancer survivors. Hawaii Med J. 2001;60(7):189, 93.

175. Gotay CC, Holup JL, Pagano I. Ethnic differences in quality of life among early breast and prostate cancer survivors. Psycho-Oncology. 2002;11(2):103-13.

176. Gotay CC, Muraoka MY. Quality of life in long-term survivors of adult-onset cancers. Journal of the National Cancer Institute. 1998;90(9):656-67.

177. Goytia EJ, Lounsbury DW, McCabe MS, Weiss E, Newcomer M, Nelson DJ, et al. Establishing a general medical outpatient clinic for cancer survivors in a public city hospital setting. Journal of General Internal Medicine. 2009;24(SUPPL. 2):S451-S5.

178. Greenbaum A, Ruckman R, Murillo M, Flores K, Rajput A, Kinney A. Interest and intent to take aspirin as chemoprevention in colorectal cancer survivors. Annals of Surgical Oncology. 2017;24(1 Supplement 1):S77.

179. Greenlee H, Gaffney AO, Aycinena AC, Koch P, Contento I, Karmally W, et al. ¡Cocinar Para Su Salud!: Randomized Controlled Trial of a Culturally Based Dietary Intervention among Hispanic Breast Cancer Survivors. Journal of the Academy of Nutrition & Dietetics. 2015;115:S42-S56.e3.

180. Greenlee H, Molmenti CL, Crew KD, Awad D, Kalinsky K, Brafman L, et al. Survivorship care plans and adherence to lifestyle recommendations among breast cancer survivors. J Cancer Surviv. 2016;10(6):956-63.

181. Hall A, Campbell H, Sanson-Fisher R, Lynagh M, d'Este C, Burkhalter R, et al. Unmet needs of Australian and Canadian haematological cancer survivors: A cross-sectional international comparative study. Psycho-Oncology. 2013;22(9):2032-8.

182. Hammond C. Against a Singular Message of Distinctness: Challenging Dominant Representations of Adolescents and Young Adults in Oncology. Journal of Adolescent & Young Adult Oncology. 2017;6(1):45-9.

183. Hamood R, Hamood H, Merhasin I, Keinan-Boker L. Work Transitions in Breast Cancer Survivors and Effects on Quality of Life. Journal of occupational rehabilitation. 2019;29(2):336-49.

184. Harju E, Roser K, Dehler S, Michel G. Health-related quality of life in adolescent and young adult cancer survivors. Supportive Care in Cancer. 2018;26(9):3099-110.

185. Hatch M, von Ehrenstein O, Wolff M, Meier K, Geduld A, Einhorn F. Using qualitative methods to elicit recall of a critical time period. Journal of Women's Health. 1999;8(2):269-77.

186. Helewa M, Lévesque P, Provencher D, Lea RH, Rosolowich V, Shapiro HM. Breast cancer, pregnancy, and breastfeeding. Journal of obstetrics and gynaecology Canada: JOGC = Journal d'obstétrique et gynécologie du Canada: JOGC. 2002;24(2):164-71.

187. Herbert SL, Wockel A, Kreienberg R, Kuhn T, Flock F, Felberbaum R, et al. To which extent do breast cancer survivors feel well informed about disease and treatment 5 years after diagnosis? Breast Cancer Research and Treatment. 2021;185(3):677-84.

188. Herbert SL, Wockel A, Kreienberg R, Kuhn T, Flock F, Felberbaum R, et al. To which extent do breast cancer survivors feel well informed about disease and treatment 5 years after diagnosis? Breast Cancer Res Treat. 2021;185(3):677-84.

189. Hilal T. Gone But Here. American Journal of Hospice & Palliative Medicine. 2020;37(8):659-60.

190. Ho SM, Law LS, Wang G-L, Shih S-M, Hsu S-H, Hou Y-C. Psychometric analysis of the Chinese version of the Posttraumatic Growth Inventory with cancer patients in Hong Kong and Taiwan. Psycho-Oncology. 2013;22(3):715-9.

191. Ho SMY, Law LSC, Wang GL, Hsu SH, Shih SM, Hou YC. Psychometric analysis of the chinese version of the post-traumatic growth inventory with cancer patients in Hong Kong and Taiwan. Proceedings of Singapore Healthcare. 2012;1):S285.

192. Hoang TM, Shin LJ, Xu S, Lu Q. Coping with breast cancer among immigrant Chinese Americans. Asian American Journal of Psychology. 2020;11(2):108-16.

193. Hodge FS, Itty TL, Cadogan MP, Martinez F, Pham A. The cultural constructs of cancer-related fatigue among American Indian cancer survivors. Supportive Care in Cancer. 2016;24(3):1235-40.

194. Holden AEC, Otto P, Gallion K, Ramirez AG. Low English language facility, depression, and cancer screening among Latina breast cancer survivors. Cancer Epidemiology Biomarkers and Prevention Conference: 7th AACR Conference on the Science of Health Disparities in Racial/Ethnic Minorities and the Medically Underserved San Antonio, TX United States Conference Publication:. 2015;24(10 SUPPL. 1).

195. Holst-Hansson A, Idvall E, Bolmsjo I, Wennick A. The narrow treatment road to survival: Everyday life perspectives of women with breast cancer from Iraq and the former Yugoslavia undergoing radiation therapy in Sweden. European journal of cancer care. 2018;27(2):e12825.

196. Hong Y, Pena-Purcell NC, Ory MG. Outcomes of online support and resources for cancer survivors: a systematic literature review. Patient Educ Couns. 2012;86(3):288-96.

197. Hsu BY, Chentsova Dutton Y, Adams IF, Gomez SL, Allen L, Huang E, et al. Talking about cancer: Explaining differences in social support among Chinese American and European American breast cancer survivors. Journal of Health Psychology. 2020;25(8):1043-56.

198. Huang RJ, Sharp N, Talamoa RO, Ji HP, Hwang JH, Palaniappan LP. One Size Does Not Fit All: Marked Heterogeneity in Incidence of and Survival from Gastric Cancer among Asian American Subgroups. Cancer Epidemiol Biomarkers Prev. 2020;29(5):903-9.

199. Hulbert-Williams NJ, Leslie M, Hulbert-Williams L, Koczwara B, Watson EK, Hall PS, et al. The Finding My Way UK Clinical Trial: Adaptation Report and Protocol for a Replication Randomized Controlled Efficacy Trial of a Web-Based Psychological Program to Support Cancer Survivors. JMIR Res Protoc. 2021;10(9):e31976.

200. Hunter J, Ussher J, Parton C, Kellett A, Smith C, Delaney G, et al. Australian integrative oncology services: A mixed-method study exploring the views of cancer survivors. BMC Complementary and Alternative Medicine. 2018;18(1) (no pagination).

201. Hunter JF, Aguilera A, Jonassaint CR, Yanez B, Low CA. Improving Health Equity with Mobile Technology. Psychosomatic Medicine. 2020;82(6):A159.

202. Hurtado-de-Mendoza A, Song M, Kigen O, Jennings Y, Nwabukwu I, Sheppard VB. Addressing cancer control needs of African-born immigrants in the US: A systematic literature review. Preventive Medicine. 2014;67:89-99.

203. Hyojin Y, Chatters L, Tsui-Sui Annie K, Saint-Arnault D, Northouse L. Factors Affecting Quality of Life for Korean American Cancer Survivors: An Integrative Review. Oncology Nursing Forum. 2016;43(3):E132-E42.

204. Hyseni F, Myderrizi A, Blanck P. Diversity and inclusion in the legal profession: disclosure of cancer and other health conditions by lawyers with disabilities and lawyers who identify as LGBTQ +. Journal of Cancer Survivorship. 2022;16(1):165-82.

205. Hyseni F, Myderrizi A, Blanck P. Diversity and inclusion in the legal profession: disclosure of cancer and other health conditions by lawyers with disabilities and lawyers who identify as LGBTQ+. Journal of cancer survivorship : research and practice. 2022;16(1):165-82.

206. Im E-O, Kim S, Lee C, Chee E, Mao JJ, Chee W. Decreasing menopausal symptoms of Asian American breast cancer survivors through a technology-based information and coaching/support program. Menopause. 2019;26(4).

207. Im E-O, Kim S, Yang YL, Chee W. The efficacy of a technology-based information and coaching/support program on pain and symptoms in Asian American survivors of breast cancer. Cancer. 2020;126(3):670-80.

208. Im EO, Ji X, Kim S, Chee E, Bao T, Mao JJ, et al. Challenges in a Technology-Based Cancer Pain Management Program Among Asian American Breast Cancer Survivors. Comput Inform Nurs. 2019;37(5):243-9.

209. Im EO, Kim S, Tsai HM, Nishigaki M, Yeo SA, Chee W, et al. Practical issues in multi-lingual research. International journal of nursing studies. 2016;54:141-9.

210. Im EO, Kim S, Xu S, Lee C, Hamajima Y, Inohara A, et al. Issues in Recruiting and Retaining Asian American Breast Cancer Survivors in a Technology-Based Intervention Study. Cancer Nursing. 2020;43(1):E22-e9.

211. Im EO, Yi JS, Kim H, Chee W. A technology-based information and coaching/support program and self-efficacy of Asian American breast cancer survivors. Res Nurs Health. 2021;44(1):37-46.

212. Jagsi R, Griffith KA, Kurian AW, Morrow M, Hamilton AS, Graff JJ, et al. Reply to S.M. Sorscher and A.B. Hafeez Bhatti. Journal of Clinical Oncology. 2015;33(35):4233.

213. Jefford M, Nolte L, Tsintziras S, Butow P, Sze M, Yiu D, et al. How is post-treatment survivorship conceptualised by people from different cultural groups? Asia-Pacific Journal of Clinical Oncology. 2014;9):158.

214. Joseph G, Nickell A, Cohen E, Burke NJ, Colen S, Lawlor K, et al. Engaging linguistically and ethnically diverse low income women in health research: A randomized controlled trial. Cancer Research Conference: San Antonio Breast Cancer Symposium, SABCS. 2017;78(4 Supplement 1).

215. Juarez G, Campesino M, Ruiz E, Glover JU, Larkey L, Koithan M, et al. Barriers in access to care among undocumented immigrant women with breast cancer. Psycho-Oncology. 2009;2):S202-S3.

216. Juarez G, Hurria A, Uman G, Ferrell B. Impact of a bilingual education intervention on the quality of life of latina breast cancer survivors. Psycho-Oncology. 2013;3):331-2.

217. Juarez G, Mayorga L. Survivorship education for latina breast cancer survivors: Empowering survivors thorugh education. Cancer Nursing. 2016;39(6 Supplement 1):S117-S8.

218. Juon HS, Choi S, Klassen A, Roter D. Impact of breast cancer screening intervention on Korean-American women in Maryland. Cancer Detection and Prevention. 2006;30(3):297-305.

219. Katapodi MC, Facione NC, Miaskowski C, Dodd MJ, Waters C. The influence of social support on breast cancer screening in a multicultural community sample. Oncology Nursing Forum. 2002;29(5):845-52.

220. Kent EE, Arora NK, Rowland JH, Bellizzi KM, Forsythe LP, Hamilton AS, et al. Health information needs and health-related quality of life in a diverse population of long-term cancer survivors. Patient Educ Couns. 2012;89(2):345-52.

221. Kessler R, Stafford D. Collaborative medicine case studies: Evidence in practice. Collaborative medicine case studies: Evidence in practice. 2008:xix, 440.

222. Kim BH, Dash C, Gomez SL, Sheppard VB, Allen L, Wang Y, et al. Associations of physical activity with physical functioning and emotional distress among non-Hispanic white and Chinese-American breast cancer survivors. Cancer Prevention Research Conference: 12th Annual AACR International Conference on Frontiers in Cancer Prevention Research National Harbor, MD United States Conference Publication:. 2013;6(11 SUPPL. 1).

223. King CJ. Racial and ethnic differences in accessing timely cancer screening and treatment services: A quantitative analysis. Dissertation Abstracts International: Section B: The Sciences and Engineering. 2014;74(10-B(E)):No Pagination Specified.

224. Kinnane N, Wiley G, Nolte L, Piper A, Evans J, Jefford M. Transforming cancer survivorship care-an Australian experience. Cancer Nursing. 2016;39(6 Supplement 1):S25.

225. Kinnane NA, Piper A, Wiley G, Nolte L, Evans J, Jefford M. Transforming Cancer Survivorship Care: An Australian Experience. Asia-Pacific Journal of Oncology Nursing. 2017;4(2):91-4.

226. Knight TG, Deal AM, Dusetzina SB, Muss HB, Choi SK, Bensen JT, et al. Financial toxicity in adults with cancer: Adverse outcomes and noncompliance. Journal of Oncology Practice. 2018;14(11):683 and e65-e73.

227. Knight TG, Deal AM, Muss HB, Dusetzina S, Choi SK, Bensen JT, et al. Financial toxicity in adults with cancer: Adverse outcomes and potential areas of intervention. Journal of Clinical Oncology Conference. 2016;34(Supplement 15).

228. Kobetz E, Menard J, Dietz N, Hazan G, Soler-Vila H, Lechner S, et al. Contextualizing the survivorship experiences of haitian immigrant women with breast cancer: Opportunities for health promotion. Oncology Nursing Forum. 2011;38(5):555-60.

229. Kooken WC, Haase JE, Russell KM. "I've been through something": poetic explorations of African American women's cancer survivorship. Western Journal of Nursing Research. 2007;29(7):896-919; discussion 20.

230. Krok-Schoen JL, Fernandez K, Unzeitig GW, Rubio G, Paskett ED, Post DM. Hispanic breast cancer patients' symptom experience and patient-physician communication during chemotherapy. Supportive Care in Cancer. 2019;27(2):697-704.

231. Kronenfeld JP, Graves KD, Penedo FJ, Yanez B. Overcoming Disparities in Cancer: A Need for Meaningful Reform for Hispanic and Latino Cancer Survivors. Oncologist. 2021;26(6):443-52.

232. Kroon LL, van Roij J, Korfage IJ, Reyners AKL, van den Beuken-van Everdingen MHJ, den Boer MO, et al. Perceptions of involvement in advance care planning and emotional functioning in patients with advanced cancer. Journal of Cancer Survivorship. 2021;15(3):380-5.

233. Kroon LL, van Roij J, Korfage IJ, Reyners AKL, van den Beuken-van Everdingen MHJ, den Boer MO, et al. Perceptions of involvement in advance care planning and emotional functioning in patients with advanced cancer. Journal of cancer survivorship : research and practice. 2021;15(3):380-5.

234. Kuehni CE, Strippoli MPF, Rueegg CS, Rebholz CE, Bergstraesser E, Grotzer M, et al. Educational achievement in Swiss childhood cancer survivors compared with the general population. Cancer. 2012;118(5):1439-49.

235. Kumar N, Perdomo B, Thornburg C, Aristizabal P. Low health literacy is associated with overconfidence in knowledge of survivorship care plans in caregivers of pediatric cancer survivors. Pediatric Blood and Cancer Conference: 52th Congress of the International Society of Paediatric Oncology, SIOP Virtual. 2020;67(SUPPL 4).

236. Kumar N, Subramanian K, Perdomo B, McDaniels-Davidson C, Thornburg C, Aristizabal P. Confidence in knowledge of survivorship care plans in pediatric cancer survivors. Pediatric Blood and Cancer Conference. 2020;67(SUPPL 2).

237. Kwok C, Ho M. Development and evaluation of a culturally sensitive support group programme for Chinese-Australian women with breast cancer: a pilot study. European Journal of Cancer Care. 2011;20(6):795-802.

238. Kwong S, Bedard A. BE ACTIVE: an Education Program for Chinese Cancer Survivors in Canada. Journal of cancer education : the official journal of the American Association for Cancer Education. 2016;31(3):605-9.

239. Kyronlahti A, Madanat-Harjuoja L, Pitkaniemi J, Rantanen M, Malila N, Taskinen M. Childhood cancer mortality and survival in immigrants: A population-based registry study in Finland. International Journal of Cancer. 2020;146(10):2746-55.

240. Lagman RA, Yoo GJ, Levine EG, Donnell KA, Lim HR. "Leaving it to God" religion and spirituality among Filipina immigrant breast cancer survivors. Journal of Religion and Health. 2014;53(2):449-60.

241. Lai-Kwon J, Heynemann S, Flore J, Dhillon H, Duffy M, Burke J, et al. Living with and beyond metastatic non-small cell lung cancer: the survivorship experience for people treated with immunotherapy or targeted therapy. Journal of Cancer Survivorship. 2021;15(3):392-7.

242. Lawler S, Maher G, Brennan M, Goode A, Reeves MM, Eakin E. Get Healthy after Breast Cancer - examining the feasibility, acceptability and outcomes of referring breast cancer survivors to a general population telephone-delivered program targeting physical activity, healthy diet and weight loss. Supportive Care in Cancer. 2017;25(6):1953-62.

243. Lawsin C, Ballard A, Dhillon H, Hobbs K, Butow P, Miller A, et al. How should we provide psycho-sexual support to cancer survivors and their partners? Assessment of intervention preferences amongst breast cancer survivors and introducing rekindle. Psycho-Oncology. 2014;3):228.

244. Le Y, Gao Z, Gomez SL, Pope Z, Dong R, Allen L, et al. Acculturation and Adherence to Physical Activity Recommendations Among Chinese American and Non-Hispanic White Breast Cancer Survivors. Journal of Immigrant & Minority Health. 2019;21(1):80-8.

245. Lebel S, Payne AY, Mah K, Irish J, Rodin G, Devins GM. Do stigma and its psychosocial impact differ between Asian-born Chinese immigrants and Western-born Caucasians with head and neck cancer? Psychology, Health & Medicine. 2016;21(5):583-92.

246. Lee H, Kivnick H. Exploring cultural life strengths in coping with cancer among elderly Korean Americans and Caucasians. Psycho-Oncology. 2010;1):S57-S8.

247. Lee HY, Jin SW. Older Korean cancer survivors' depression and coping: Directions toward culturally competent interventions. Journal of Psychosocial Oncology. 2013;31(4):357-76.

248. Lee J. "joining in": The subtleties of culture and race in the psychosocial experience of diverse patients in psychotherapy with a racially diverse therapist. Psycho-Oncology. 2020;29(Supplement 1):59.

249. Lee S, Chen L, Ma GX, Fang CY, Oh Y, Scully L. Challenges and Needs of Chinese and Korean American Breast Cancer Survivors: In-Depth Interviews. N. 2013;6(1):1-8.

250. Lee S, Ma GX, Fang CY, Chen L, Youngsuk O. Catering to the needs of young and older Asian American breast cancer survivors: In-depth interviews. Cancer Epidemiology Biomarkers and Prevention Conference: American Association for Cancer Research, AACR International Conference on the Science of Cancer Health Disparities. 2011;20(10 Meeting Abstracts).

251. Leimanis ML. An exploration of Latvian immigrants' cancer experience and implications for supportive interventions. Treating vulnerable populations of cancer survivors: A biopsychosocial approach. 2016:37-54.

252. Leng J, Lee T, Sarpel U, Lau J, Li Y, Cheng C, et al. Identifying the informational and psychosocial needs of Chinese immigrant cancer patients: a focus group study. Supportive Care in Cancer. 2012;20(12):3221-9.

253. Leng J, Lui F, Chen A, Huang X, Breitbart W, Gany F. Adapting Meaning-Centered Psychotherapy in Advanced Cancer for the Chinese Immigrant Population. Journal of Immigrant and Minority Health. 2018;20(3):680-6.

254. Levi RB, Drotar D. Preliminary translation and cultural adaptation of health utilities index questionnaires for application in Argentina. International Journal of Cancer. 1999(SUPPL. 12):119-24.

255. Levine E, Lee J, Yoo G. Breast cancer among chinese immigrant women: Understanding unmet needs. Psycho-Oncology. 2013;2):49-50.

256. Liebschutz J, Battaglia T, Finley E, Averbuch T. Disclosing intimate partner violence to health care clinicians - What a difference the setting makes: A qualitative study. BMC Public Health. 2008;8.

257. Lim BT, Butow P, Mills J, Miller A, Pearce A, Goldstein D. Challenges and perceived unmet needs of Chinese migrants affected by cancer: Focus group findings. Journal of Psychosocial Oncology. 2019;37(3):383-97.

258. Lim BT, Mills J, Miller A, Butow P, Goldstein D, Pearce A. Cancer information and support webinar series for the Australian Chinese community. Psycho-Oncology. 2016;2):46.

259. Lim J-W, Baik OM, Ashing-Giwa KT. Cultural health beliefs and health behaviors in Asian American breast cancer survivors: A mixed-methods approach. Oncology Nursing Forum. 2012;39(4):388-97.

260. Lim J-w, Townsend A. Cross-Ethnicity Measurement Equivalence of Family Coping for Breast Cancer Survivors. Research on Social Work Practice. 2012;22(6):689-703.

261. Lim J-w, Yi J. The effects of religiosity, spirituality, and social support on quality of life: A comparison between Korean American and Korean breast and gynecologic cancer survivors. Oncology Nursing Forum. 2009;36(6):699-708.

262. Lim J-w, Yi J, Zebrack B. Acculturation, social support, and quality of life for Korean immigrant breast and gynecological cancer survivors. Ethnicity & Health. 2008;13(3):243-60.

263. Lim JW. The role of culture and ethnicity in the adjustment to gynecological cancer. Current Women's Health Reviews. 2011;7(4):379-90.

264. Lim JW, Yi J. The effects of religiosity, spirituality, and social support on quality of life: a comparison between Korean American and Korean breast and gynecologic cancer survivors. Oncology nursing forum. 2009;36(6):699-708.

265. Lim JW, Yi J, Zebrack B. Acculturation, social support, and quality of life for Korean immigrant breast and gynecological cancer survivors. Ethnicity and Health. 2008;13(3):243-60.

266. Lindahl M, Addington SV, Winther JF, Schmiegelow K, Andersen KK. Socioeconomic factors and ninth grade school performance in childhood leukemia and CNS tumor survivors. JNCI Cancer Spectrum. 2018;2(1) (no pagination).

267. Liu S, Smith EC, McCartney A, Basgaran A, Balaratnam K, Catherine Brown M, et al. Assessment of language needs and resource use among Canadian immigrant patients with cancer. Journal of Clinical Oncology Conference. 2018;36(30 Supplement 1).

268. Liu SY, Lu L, Balaratnam K, Pringle D, Mahler M, Niu C, et al. Impact of immigration status on health behaviors and perceptions in cancer survivors. Journal of Clinical Oncology Conference. 2018;36(7).

269. Liu SY, Lu L, Pringle D, Mahler M, Niu C, Charow R, et al. Impact of immigration status on health behaviors and perceptions in cancer survivors. Cancer Medicine. 2019;8(5):2623-35.

270. Lockhart JS, Oberleitner MG, Nolfi DA. The Hispanic/Latino immigrant cancer survivor experience in the United States: A scoping review. Blutalkohol. 2018;2(4):119-60.

271. Lockhart JS, Oberleitner MG, Nolfi DA. The Hispanic/Latino Immigrant Cancer Survivor Experience in the United States: A Scoping Review. Annual Review of Nursing Research. 2019;37(1):119-60.

272. Lockhart JS, Oberleitner MG, Nolfi DA. The Asian Immigrant Cancer Survivor Experience in the United States: A Scoping Review of the Literature. Cancer Nursing. 2020;43(3):177-99.

273. Lopez G. The Latina breast cancer survivor's cultural baggage: A young immigrant, survivor, and advocate's perspective. Breast Diseases. 2013;24(4):317-8.

274. Lopez MM. Concerns, coping, and quality of life of Mexican American breast cancer survivors. 2012(Ph.D.):201 p- p.

275. Lopez-Class M, Perret-Gentil M, Kreling B, Caicedo L, Mandelblatt J, Graves KD. Quality of life among immigrant Latina breast cancer survivors: realities of culture and enhancing cancer care. Journal of cancer education : the official journal of the American Association for Cancer Education. 2011;26(4):724-33.

276. Lu Q, Chu Q, Warmoth K, Young N, Young L, Loh A, et al. Understanding the unique experience among Chinese immigrant breast cancer survivors. Cancer Epidemiology Biomarkers and Prevention Conference: 11th AACR Conference on the Science of Cancer Health Disparities in Racial/Ethnic Minorities and the Medically Underserved New Orleans, LA United States. 2020;29(6 SUPPL 1).

277. Lu Q, Kagawa-Singer M, Young L, Loh A, Chen M. To test the cultural sensitivity, feasibility, and health benefits of an intervention of expressive writing among Chinese-speaking breast cancer survivors. Journal of Cancer Education. 2009;1):68.

278. Lu Q, Kagawa-Singer M, Young L, Loh A, Chen M. Learning from success and challenge in expressive writing intervention among Chinese breast cancer survivors. Psycho-Oncology. 2010;1):S59-S60.

279. Lu Q, Man J, Yeung N, You J, Young L, Loh A. Sources of distress and culturally sensitive interventions to reduce distress among Chinese-speaking breast cancer survivors. Psycho-Oncology. 2012;1):36.

280. Lu Q, Warmoth K, Chen L, Wu CS, Chu Q, Li Y, et al. A Culturally Sensitive Social Support Intervention for Chinese American Breast Cancer Survivors (Joy Luck Academy): Protocol for a Randomized Controlled Trial. JMIR Res Protoc. 2021;10(9):e30950.

281. Lu Q, Yeung NC, You J, Dai J. Using expressive writing to explore thoughts and beliefs about cancer and treatment among Chinese American immigrant breast cancer survivors. Psycho-Oncology. 2016;25(11):1371-4.

282. Lu Q, You J, Man J, Loh A, Young L. Evaluating a Culturally Tailored Peer-Mentoring and Education Pilot Intervention Among Chinese Breast Cancer Survivors Using a Mixed-Methods Approach. Oncology Nursing Forum. 2014;41(6):629-37.

283. Lu Q, Young L, Loh A, Kagawa-Singer M, Chen M, Zheng D. Lessons learned from an expressive writing intervention among minority breast cancer survivors using community-based participatory approach and mixed methods. Psycho-Oncology. 2011;1):29-30.

284. Lu Q, Zheng D, Young L, Kagawa-Singer M, Loh A. A pilot study of expressive writing intervention among Chinese-speaking breast cancer survivors. Health psychology : official journal of the Division of Health Psychology, American Psychological Association. 2012;31(5):548-51.

285. Luckett T, Goldstein D, Butow PN, Gebski V, Aldridge LJ, McGrane J, et al. Psychological morbidity and quality of life of ethnic minority patients with cancer: A systematic review and meta-analysis. The Lancet Oncology. 2011;12(13):1240-8.

286. Lui F, Chen A, Huang X, Gany F, Breitbart W, Leng J. Adapting meaning-centered psychotherapy in advanced cancer for the Chinese immigrant population. Psycho-Oncology. 2015;2):350.

287. Lutzker JR, Merrick J. Applied public health: Examining multifaceted social or ecological problems and child maltreatment. Applied public health: Examining multifaceted social or ecological problems and child maltreatment. 2012:xiii, 251.

288. Lyson HC, Haggstrom D, Bentz M, Obeng-Gyasi S, Dixit N, Sarkar U. Communicating Critical Information to Cancer Survivors: an Assessment of Survivorship Care Plans in Use in Diverse Healthcare Settings. Journal of Cancer Education. 2021;36(5):981-9.

289. Mahendran R, Liu J, Kuparasundram S, Griva K. Validation of the English and simplified Mandarin versions of the Fear of Progression Questionnaire - Short Form in Chinese cancer survivors. BMC psychology. 2020;8(1):10.

290. Majhail NS, Murphy E, Laud P, Preussler JM, Denzen EM, Abetti B, et al. Randomized controlled trial of individualized treatment summary and survivorship care plans for hematopoietic cell transplantation survivors. Haematologica. 2019;104(5):1084-92.

291. Majhail NS, Murphy EA, Laud P, Preussler J, Denzen E, Adams A, et al. Individualized treatment summaries and survivorship care plans (SCPs) for hematopoietic cell transplant (HCT) survivors reduces cancer treatment distress in a randomized, multicenter study. Blood Conference: 59th Annual Meeting of the American Society of Hematology, ASH. 2017;130(Supplement 1).

292. Manglona RD, Robert S, Isaacson LSN, Garrido M, Henrich FB, Santos LS, et al. Promoting breast cancer screening through storytelling by chamorro cancer survivors. Californian Journal of Health Promotion. 2010;8(2):90-5.

293. Marquis A, Strippoli MPF, Spycher BD, Rebholz CE, Von Der Weid NX, Kuehni CE. Paracetamol, nonsteroidal anti-inflammatory drugs, and risk of asthma in adult survivors of childhood cancer. Journal of Allergy and Clinical Immunology. 2011;127(1):270-2.

294. Martinez Tyson D, Medina-Ramirez P, Vázquez-Otero C, Gwede CK, Bobonis M, McMillan SC. Cultural adaptation of a supportive care needs measure for Hispanic men cancer survivors. Journal of Psychosocial Oncology. 2018;36(1):113-31.

295. Maskarinec G, Pagano I, Lurie G, Bantum E, Gotay CC, Issell BF. Factors affecting survival among women with breast cancer in hawaii. Journal of Women's Health (15409996). 2011;20(2):231-7.

296. Matthew A. Core principles of sexual health treatments in cancer for men. Current opinion in supportive and palliative care. 2016;10(1):38-43.

297. McBride ML, Groome P, Turner D, Jorgensen M, Kendell C, Porter G, et al. Using Canadian administrative data to evaluate primary and oncology care of breast cancer patients post-treatment: Subset of the CanIMPACT Study. Journal of Clinical Oncology Conference. 2016;34(3 SUPPL. 1).

298. McDougall JA, Banegas MP, Wiggins C, Rajput A, Chiu VK, Flores KG, et al. Disparities in treatment-related financial hardship and adherence to surveillance colonoscopy guidelines in ethnically, linguistically, and geographically diverse colorectal cancer survivors. Cancer Epidemiology Biomarkers and Prevention Conference: 10th AACR Conference on the Science of Cancer Health Disparities in Racial/Ethnic Minorities and the Medically Underserved Atlanta, GA United States. 2018;27(7 Supplement).

299. McGrane J, Butow P, Sze M, Eisenbruch M, Goldstein D, King M. Assessing the invariance of a culturally competent multi-lingual unmet needs survey for immigrant and Australian-born cancer patients: A Rasch analysis. Psycho-Oncology. 2014;3):153.

300. McGrane J, King M, Butow P. Bridging cultures requires culturally invariant assessment: Investigating the cross-cultural invariance of psychosocial outcomes assessments using the rasch model. Psycho-Oncology. 2011;2):69-70.

301. McKinley CE, Roh S, Lee Y-S, Liddell J. Family: The bedrock of support for American Indian women cancer survivors. Family & Community Health: The Journal of Health Promotion & Maintenance. 2020;43(3):246-54.

302. McMullen CK, Bulkley JE, Altschuler A, Wendel CS, Grant M, Hornbrook MC, et al. Greatest challenges of rectal cancer survivors: Results of a population-based survey. Diseases of the Colon and Rectum. 2016;59(11):1019-27.

303. McMullin JM, Taumoepeau L, Talakai M, Kivalu F, Hubbell FA. Tongan perceptions of cancer. Cancer Detection and Prevention. 2008;32(1 SUPPL.):29-36.

304. Medeiros EA, Castañeda SF, Gonzalez P, Rodríguez B, Buelna C, West D, et al. Health-Related Quality of Life Among Cancer Survivors Attending Support Groups. Journal of Cancer Education. 2015;30(3):421-7.

305. Merrick J. Public health yearbook, 2010. Public health yearbook, 2010. 2012:xxxiii, 716.

306. Merten M, Wald M, Harris A, Johnson H. Implementation of survivorship care plans in a thyroid cancer population. Thyroid. 2018;28(Supplement 1):A36-A7.

307. Moadel A. Psycho-educational and spiritual interventions for low income cancer patients: Results of a randomized versus patient preference trial. Psycho-Oncology. 2011;1):30-1.

308. Moadel AB, Shah C, Wylie-Rosett J, Harris MS, Patel SR, Hall CB, et al. Randomized controlled trial of yoga among a multiethnic sample of breast cancer patients: effects on quality of life. J Clin Oncol. 2007;25(28):4387-95.

309. Morawa E, Erim Y. Depressive complaints and utilization of mental health services: Comparison of adult cancer survivors of different ethnic origin. Journal of Psychosomatic Research. 2020;130 (no pagination).

310. Muliira RS, Salas AS, O'Brien B. Quality of Life among Female Cancer Survivors in Africa: An Integrative Literature Review. Asia-Pacific Journal of Oncology Nursing. 2017;4(1):6-17.

311. Nápoles AM, Ortíz C, Santoyo-Olsson J, Stewart AL, Gregorich S, Lee HE, et al. Nuevo Amanecer: Results of a Randomized Controlled Trial of a Community-Based, Peer-Delivered Stress Management Intervention to Improve Quality of Life in Latinas With Breast Cancer. American Journal of Public Health. 2015;105(S3):e55-e63.

312. Nápoles AM, Ortiz C, Santoyo-Olsson J, Stewart AL, Lee HE, Duron Y, et al. Post-Treatment Survivorship Care Needs of Spanish-speaking Latinas with Breast Cancer. J Community Support Oncol. 2017;15(1):20-7.

313. Napoles AM, Santoyo-Olsson J, Chacon L, Stewart AL, Dixit N, Ortiz C. Feasibility of a Mobile Phone App and Telephone Coaching Survivorship Care Planning Program Among Spanish-Speaking Breast Cancer Survivors. JMIR Cancer. 2019;5(2):e13543.

314. Nápoles AM, Santoyo-Olsson J, Stewart AL, Ortiz C, Samayoa C, Torres-Nguyen A, et al. Nuevo Amanecer-II: Results of a randomized controlled trial of a community-based participatory, peer-delivered stress management intervention for rural Latina breast cancer survivors. Psycho-Oncology. 2020;29(11):1802-14.

315. Nápoles-Springer AM, Ortíz C, O'Brien H, Díaz-Méndez M, Pérez-Stable EJ. Use of cancer support groups among Latina breast cancer survivors. J Cancer Surviv. 2007;1(3):193-204.

316. Naranjo LE, Dirksen SR. The recruitment and participation of Hispanic women in nursing research: a learning process. Public Health Nursing. 1998;15(1):25-9.

317. Natori A, Sookdeo VD, Koru-Sengul T, Schlumbrecht M, Calfa C, MacIntyre J, et al. Predictors of adherence to patient reported outcomes and psychosocial needs questionnaire in a culturally diverse ambulatory oncology setting: The My Wellness Check Program. Journal of Clinical Oncology Conference. 2021;39(28 SUPPL).

318. Nevarez L, Hovick SR, Enard KR, Lloyd SM, Kahlor LA. Race/Ethnic Variations in Predictors of Health Consciousness Within the Cancer Prevention Context. American journal of health promotion : AJHP. 2020;34(7):740-6.

319. Nguyen T. Posttraumatic growth in pediatric cancer patients: Differences between Asian and western populations and the role of psychologists in facilitating it. Dissertation Abstracts International: Section B: The Sciences and Engineering. 2022;83(4-B):No Pagination Specified.

320. Nickell A, Burke NJ, Cohen E, Caprio M, Joseph G. Educating low-SES and LEP survivors about breast cancer research: pilot test of the Health Research Engagement Intervention. Journal of cancer education : the official journal of the American Association for Cancer Education. 2014;29(4):746-52.

321. Nickell A, Cohen E, Stewart S, Cheng JKY, Lawlor K, Colen S, et al. Engaging linguistically and ethnically diverse low income women in health research. Cancer Epidemiology Biomarkers and Prevention Conference: 9th AACR Conference on the Science of Cancer Health Disparities in Racial/Ethnic Minorities and the Medically Underserved Fort Lauderdale, FL United States. 2017;26(2 Supplement 1).

322. Nickell A, Stewart SL, Burke NJ, Guerra C, Cohen E, Lawlor C, et al. Engaging limited English proficient and ethnically diverse low-income women in health research: A randomized trial of a patient navigator intervention. Patient Education and Counseling. 2019;102(7):1313-23.

323. Nolan TS, Bell AM, Chan YN, Leak Bryant A, Bissram JS, Hirschey R. Use of Video Education Interventions to Increase Racial and Ethnic Diversity in Cancer Clinical Trials: A Systematic Review. Worldviews on Evidence-Based Nursing. 2021;18(5):302-9.

324. O'Callaghan C, Dharmagesan G, Roy J, Dharmagesan V, Loukas P, Harris-Roxas B. Enhancing equitable access to cancer information for culturally and linguistically diverse (CALD) communities to complement beliefs about cancer prognosis and treatment. Supportive Care in Cancer. 2021;29(10):5957-65.

325. O'Callaghan C, Dharmagesan GG, Roy J, Dharmagesan V, Loukas P, Harris-Roxas B. Enhancing equitable access to cancer information for culturally and linguistically diverse (CALD) communities to complement beliefs about cancer prognosis and treatment. Supportive Care in Cancer. 2021;29(10):5957-65.

326. O'Callaghan C, Schofield P, Butow P, Nolte L, Price M, Tsintziras S, et al. "I might not have cancer if you didn't mention it": a qualitative study on information needed by culturally diverse cancer survivors. Supportive Care in Cancer. 2016;24(1):409-18.

327. O’Callaghan C, Schofield P, Butow P, Nolte L, Price M, Tsintziras S, et al. “I might not have cancer if you didn’t mention it”: a qualitative study on information needed by culturally diverse cancer survivors. Supportive Care in Cancer. 2016;24(1):409-18.

328. Ochoa CY, Miller KA, Baezconde-Garbanati L, Slaughter RI, Hamilton AS, Milam JE. Parental Cancer-related Information Seeking, Health Communication and Satisfaction with Medical Providers of Childhood Cancer Survivors: Differences by Race/Ethnicity and Language Preference. Journal of Health Communication. 2021;26(2):83-91.

329. Okubo R, Wada S, Shimizu Y, Tsuji K, Hanai A, Imai K, et al. Expectations of and recommendations for a cancer survivorship guideline in Japan: A literature review of guidelines for cancer survivorship. Japanese Journal of Clinical Oncology. 2019;49(9):812-22.

330. Olagunju TO, Liu Y, Liang LJ, Stomber JM, Griggs JJ, Ganz PA, et al. Disparities in the survivorship experience among Latina survivors of breast cancer. Cancer. 2018;124(11):2373-80.

331. Ortiz TM. Childhood cancer survivorship: Understanding the impact on Asian-American families. Dissertation Abstracts International: Section B: The Sciences and Engineering. 2013;74(2-B(E)):No Pagination Specified.

332. Osann K, Wenzel L, Dogan A, Hsieh S, Chase DM, Sappington S, et al. Recruitment and retention results for a population-based cervical cancer biobehavioral clinical trial. Gynecologic Oncology. 2011;121(3):558-64.

333. Owens OL, Smith KN, Beer JM, Gallerani DG, McDonnell KK. A qualitative cultural sensitivity assessment of the Breathe Easier mobile application for lung cancer survivors and their families. Oncology Nursing Forum. 2020;47(3):331-41.

334. Palmer NRA, Gregorich S, Livaudais-Toman J, Kaplan C. Racial and ethnic disparities in prostate cancer survivors' perceived engagement in treatment decision making. Cancer Epidemiology Biomarkers and Prevention Conference: 9th AACR Conference on the Science of Cancer Health Disparities in Racial/Ethnic Minorities and the Medically Underserved Fort Lauderdale, FL United States. 2017;26(2 Supplement 1).

335. Pan M, Lee ATC, Ahmed E. Development of a Web-based community cancer survivorship program. Journal of Clinical Oncology Conference: ASCO Annual Meeting. 2011;29(15 SUPPL. 1).

336. Pan T, Malcarne V, Branz P, Fager M, Sadler GR. Signs of hope: A cancer education program for the deaf community. Psycho-Oncology. 2013;2):63-4.

337. Pare-Blagoev EJ, Ruble K, Jacobson LA. Tools of the trade to address schooling related communication needs after childhood cancer: A mini-review with consideration of health disparity concerns. Seminars in Oncology. 2020;47(1):65-72.

338. Park S-Y, Kang M, Shvetsov YB, Setiawan VW, Boushey CJ, Haiman CA, et al. Diet quality and all-cause and cancer-specific mortality in cancer survivors and non-cancer individuals: the Multiethnic Cohort Study. European Journal of Nutrition. 2022;61(2):925-33.

339. Patel G, Harcourt D, Rumsey N, Naqvi H. The experiences of breast cancer in gujarati speaking Indian women. Psycho-Oncology. 2012;2):7.

340. Patel K, Wall K, Bott NT, Katonah DG, Koopman C. A qualitative investigation of the effects of psycho-spiritual integrative therapy on breast cancer survivors' experience of paradox. Journal of Religion and Health. 2015;54(1):253-63.

341. Patel KN. Working with paradoxes: Moving from dialectical opposition to interdependent growth in culturally diverse female cancer survivors. Dissertation Abstracts International: Section B: The Sciences and Engineering. 2013;74(3-B(E)):No Pagination Specified.

342. Pearl J. How do I encourage physical activity and health promotion behaviors when providing culturally sensitive care to cancer survivors? Clinical Journal of Oncology Nursing. 2021;25(6):736-.

343. Pedro L. Theory derivation: adaptation of a contextual model of health related quality of life to rural cancer survivors. Online Journal of Rural Nursing & Health Care. 2010;10(1):80-95.

344. Peterson S, Hovick S, Burton-Chase A, Ba F, Basen-Engquist K, Fisch M, et al. Preferences for eHealth technology in meeting the health information needs of underserved cancer survivors. Psycho-Oncology. 2014;3):73-4.

345. Peterson SK, Basen-Engquist K, Demark-Wahnefried W, Prokhorov AV, Shinn EH, Martch SL, et al. Feasibility of using home-based mobile sensors for remote patient monitoring in cancer care and prevention. Journal of Clinical Oncology Conference. 2014;32(15 SUPPL. 1).

346. Pierce L. Asco's commitment to addressing equity, diversity, and inclusion of black cancer patients and survivors. JCO Oncology Practice. 2021;17(5):255-7.

347. Powell R, Butow P, Bu S, Charles C, Gafni A, Lam W, et al. "Good things come in three's?" patient, family, and health professional experiences of triadic communication and decision-making in cancer consultations. Psycho-Oncology. 2011;2):98-9.

348. Pratt-Chapman M, Simon MA, Patterson AK, Risendal BC, Patierno S. Survivorship navigation outcome measures: a report from the ACS patient navigation working group on survivorship navigation. Cancer. 2011;117(15 Suppl):3573-82.

349. Pritchard-Jones K, Pieters R, Reaman GH, Hjorth L, Downie P, Calaminus G, et al. Sustaining innovation and improvement in the treatment of childhood cancer: Lessons from high-income countries. The Lancet Oncology. 2013;14(3):e95-e103.

350. Prochaska JJ, Coughlin SS, Lyons EJ. Social Media and Mobile Technology for Cancer Prevention and Treatment. American Society of Clinical Oncology educational book. 2017;American Society of Clinical Oncology. Annual Meeting. 37:128-37.

351. Quach T, Nuru-Jeter A, Morris P, Allen L, Shema SJ, Winters JK, et al. Experiences and perceptions of medical discrimination among a multiethnic sample of breast cancer patients in the Greater San Francisco Bay Area, California. American journal of public health. 2012;102(5):1027-34.

352. Quinn G, Wells K, Antonia T, Antolino P, Lopez N, Gonzalez L, et al. Evaluating Spanish audiovisual materials about cancer clinical trials: Improving awareness among Hispanic patients and families. Cancer Epidemiology Biomarkers and Prevention Conference: American Association for Cancer Research, AACR International Conference on the Science of Cancer Health Disparities. 2011;20(10 Meeting Abstracts).

353. Rajeev P, Bach SC, Chessky A, Goddard B, Torresan M, Ades V. Knowledge and Use of Contraception among a Population of Female Survivors of Torture. Obstetrics and Gynecology Conference: 67th Annual Clinical and Scientific Meeting of the American College of Obstetricians and Gynecologists Nashville, TN United States. 2019;133(SUPPL 1).

354. Reilly R, Micklem J, Yerrell P, Banham D, Morey K, Stajic J, et al. Aboriginal experiences of cancer and care coordination: Lessons from the Cancer Data and Aboriginal Disparities (CanDAD) narratives. Health expectations : an international journal of public participation in health care and health policy. 2018;21(5):927-36.

355. Reilly R, Micklem J, Yerrell P, Banham D, Morey K, Stajic J, et al. Aboriginal experiences of cancer and care coordination: Lessons from the Cancer Data and Aboriginal Disparities (CanDAD) narratives. Health Expect. 2018;21(5):927-36.

356. Riccetti N, Felberbaum R, Flock F, Kuhn T, Leinert E, Schwentner L, et al. Financial difficulties in breast cancer survivors with and without migration background in Germany-results from the prospective multicentre cohort study BRENDA II. Supportive Care in Cancer. 2022;04:04.

357. Riccetti N, Werner AM, Ernst M, Hempler I, Singer S. Information and supportive care needs of migrants and ethnic minorities with cancer-an umbrella review. [German]. Onkologe. 2020;26(10):957-65.

358. Riccetti N, Werner AM, Ernst M, Hempler I, Singer S. Migrants and ethnic minorities with cancer: an umbrella review on their information and supportive care needs. Onkologe. 2021;27(Supplement 2):133-44.

359. Ritwik P. Dental care for patients with childhood cancers. Ochsner Journal. 2018;18(4):351-7.

360. Rosales M, Ashing-Giwa K. Young breast cancer survivors' quality of care. Psycho-Oncology. 2013;2):104-5.

361. Rossi A, Garber CE, Kaur G, Xue X, Goldberg GL, Nevadunsky NS. Physical activity-related differences in body mass index and patient-reported quality of life in socioculturally diverse endometrial cancer survivors. Supportive Care in Cancer. 2017;25(7):2169-77.

362. Ruddy KJ, Greaney ML, Sprunck-Harrild K, Meyer ME, Emmons KM, Partridge AH. A qualitative exploration of supports and unmet needs of diverse young women with breast cancer. J. 2015;13(9):323-9.

363. Rueegg CS, von der Weid NX, Rebholz CE, Michel G, Zwahlen M, Grotzer M, et al. Daily physical activities and sports in adult survivors of childhood cancer and healthy controls: A population-based questionnaire survey. PLoS ONE. 2012;7(4) (no pagination).

364. Ruhl C, Moran B. The clinical content of preconception care: preconception care for special populations. American Journal of Obstetrics and Gynecology. 2008;199(6 SUPPL. B):S384-S8.

365. Rushton M, Pudwell J, Wei S, Richardson H, Velez M. in Ontario, Canada: a population-based study. Breast. 2021;56(Supplement 1):S79.

366. Russell L, Ugalde A, White V, Pitcher M, Jorgenssen S, Whitfield K, et al. Relevance of mindfulness practices for culturally and linguistically diverse cancer populations. Psycho-Oncology. 2019;28(11):2250-2.

367. Safi S, Ghahate D, Bobelu J, Wandinger-Ness A, Faber T, Mishra S, et al. Assessing knowledge and perceptionsabout cancer among American Indians of Zuni Pueblo,New Mexico. Cancer Epidemiology Biomarkers and Prevention Conference: 12th AACR Conference on the Science of Cancer Health Disparities in Racial/Ethnic Minorities and the Medically Underserved San Francisco, CA United States. 2020;29(6 SUPPL 2).

368. Sammarco A, Konecny LM. Quality of life, social support, and uncertainty among Latina and Caucasian breast cancer survivors: A comparative study. Oncology Nursing Forum. 2010;37(1):93-9.

369. Sanders LD, Wilmoth MC, Lowry B. Overcoming: breast cancer and its effect on intimacy in middle aged African-American women. Journal of National Black Nurses Association. 2004;15(2):32-9.

370. Santoyo-Olsson J, Stewart AL, Samayoa C, Palomino H, Urias A, Gonzalez N, et al. Translating a stress management intervention for rural Latina breast cancer survivors: The Nuevo Amanecer-II. PLOS ONE. 2019;14(10):e0224068.

371. Schliemann D, Su TT, Paramasivam D, Somasundaram S, Ibrahim Tamin NSB, Dahlui M, et al. The systematic cultural adaptation of a UK public health cancer awareness raising programme for Malaysia: The Be Cancer Alert Campaign. Translational Behavioral Medicine. 2019;9(6):1087-99.

372. Schwartz NA, von Glascoe CA. The Body in the Mirror: Breast Cancer, Liminality and Borderlands. Medical anthropology. 2021;40(1):64-78.

373. Scott DL, Levine SB. Understanding gay and lesbian life. Handbook of clinical sexuality for mental health professionals , 2nd ed. 2010:351-68.

374. Seay J, Hicks A, Markham MJ, Schlumbrecht M, Bowman M, Woodard J, et al. Developing a web-based LGBT cultural competency training for oncologists: The COLORS training. Patient Education and Counseling. 2019;102(5):984-9.

375. Seo JY, Strauss SM. Association of Cancer History and Health Care Utilization Among Female Immigrants Using NHANES 2007-2016 Data. Journal of Immigrant and Minority Health. 2020;22(6):1208-16.

376. Shaw MD, Coggin C. Using a Delphi technique to determine the needs of African American breast cancer survivors. Health Promotion Practice. 2008;9(1):34-44.

377. Sheth GR, Cranmer LD, Smith BD, Grasso-LeBeau L, Lang JE. Radiation-induced sarcoma of the breast: A systematic review. Oncologist. 2012;17(3):405-18.

378. Shimoda S, De Camargo B, Horsman J, Furlong W, Lopes LF, Seber A, et al. Translation and cultural adaptation of Health Utilities Index (HUI) Mark 2 (HUI2) and Mark 3 (HUI3) with application to survivors of childhood cancer in Brazil. Quality of Life Research. 2005;14(5):1407-12.

379. Singh-Carlson S. Developing respectful and effective communication within clinician-client relationship as well as the family to patients' meet their need for healthy support system. Psycho-Oncology. 2011;1):53-4.

380. Sleight AG, Ramirez CN, Miller KA, Milam JE. Hispanic Orientation and Cancer-Related Knowledge in Childhood Cancer Survivors. Journal of Adolescent and Young Adult Oncology. 2019;8(3):363-7.

381. Smith-Gagen J, Ang A, Carrillo JE, Perez-Stable E. Is a lack of quality follow-up care related to shorter survival in latino cancer survivors? American Journal of Epidemiology. 2011;11):S313.

382. Solomon FM, Eberl-Lefko AC, Michaels M, Macario E, Tesauro G, Rowland JH. Development of a linguistically and culturally appropriate booklet for Latino cancer survivors: lessons learned. Health Promotion Practice. 2005;6(4):405-13.

383. Song L, Hamilton JB, Moore AD. Patient-healthcare provider communication: Perspectives of African American cancer patients. Health Psychology. 2012;31(5):539-47.

384. Sousa M, Moreira H, Melo C, Canavarro MC, Barreto Carvalho C. The mediating role of unmet needs in the relationship between displacement and psychological adjustment: A study of cancer survivors from a Portuguese island region. European Journal of Oncology Nursing. 2021;52:N.PAG-N.PAG.

385. Sparks L, Mittapalli K. To know or not to know: the case of communication by and with older adult Russians diagnosed with cancer. Journal of Cross-Cultural Gerontology. 2004;19(4):383-403.

386. Stephens M, Halcomb E, Dewing J. Living on: an exploration of healthful cancer survivorship among grey nomads. Australian Journal of Cancer Nursing. 2018;19(1):19-24.

387. Stolk Y, Kaplan I, Szwarc J. Clinical use of the Kessler psychological distress scales with culturally diverse groups. International Journal of Methods in Psychiatric Research. 2014;23(2):161-83.

388. Su M, Hua X, Wang J, Yao N, Zhao D, Liu W, et al. Health-related quality of life among cancer survivors in rural China. Quality of Life Research. 2019;28(3):695-702.

389. Subramanian K, Jaimes-Alvarez L, Proudfoot J, Thornburg C, Aristizabal P. Confidence in survivorship care in parents of young cancer survivors: Does health literacy matter? Pediatric Blood and Cancer. 2017;64(Supplement 1):S74.

390. Sungur H, Yilmaz NG, Chan BMC, van den Muijsenbergh METC, van Weert JCM, Schouten BC. Development and Evaluation of a Digital Intervention for Fulfilling the Needs of Older Migrant Patients with Cancer: User-Centered Design Approach. Journal of Medical Internet Research. 2020;22(10) (no pagination).

391. Szecket N, Medin G, Furlong WJ, Feeny DH, Barr RD, Depauw S. Preliminary translation and cultural adaptation of Health Utilities Index questionnaires for application in Argentina. International journal of cancer. 1999;Supplement = Journal international du cancer. Supplement. 12:119-24.

392. Tadmor T, Liphshitz I, Silverman B, Polliack A. Incidence and epidemiology of non-Hodgkin lymphoma and risk of second malignancy among 22 466 survivors in Israel with 30 years of follow-up. Hematological Oncology. 2017;35(4):599-607.

393. Tan L, Gallego G, Nguyen TTC, Bokey L, Reath J. Perceptions of shared care among survivors of colorectal cancer from non-English-speaking and English-speaking backgrounds: a qualitative study. BMC family practice. 2018;19(1):134.

394. Tenenbaum G, Eklund RC. Handbook of sport psychology., 3rd ed. Handbook of sport psychology. 2007:xxi, 937.

395. Tervonen HE, Aranda S, Roder D, You H, Walton R, Morrell S, et al. Cancer survival disparities worsening by socio-economic disadvantage over the last 3 decades in new South Wales, Australia. BMC Public Health. 2017;17(1):691.

396. Tin Tin S, Elwood JM, Brown C, Sarfati D, Campbell I, Scott N, et al. Ethnic disparities in breast cancer survival in New Zealand: which factors contribute? BMC Cancer. 2018;18(1):58.

397. Tisnado DM, Mendez-Luck C, Metz J, Peirce K, Montano B. Perceptions of Survivorship Care among Latina Women with Breast Cancer in Los Angeles County. Public health nursing (Boston, Mass). 2017;34(2):118-29.

398. Tobin J, Allem J-P, Slaughter R, Unger JB, Hamilton AS, Milam JE. Posttraumatic growth among childhood cancer survivors: Associations with ethnicity, acculturation, and religious service attendance. Journal of Psychosocial Oncology. 2018;36(2):175-88.

399. Tobin J, Cockburn M, Finch BK, Hamilton AS, Milam JE. Multilevel factors associated with depressive symptoms among long-term survivors of childhood cancer. Cancer Epidemiology Biomarkers and Prevention Conference: 12th AACR Conference on the Science of Cancer Health Disparities in Racial/Ethnic Minorities and the Medically Underserved San Francisco, CA United States. 2020;29(6 SUPPL 2).

400. Tompkins C, Scanlon K, Scott E, Ream E, Harding S, Armes J. Survivorship care and support following treatment for breast cancer: a multi-ethnic comparative qualitative study of women's experiences. BMC health services research. 2016;16(1):401.

401. Torp S, Paraponaris A, Van Hoof E, Lindbohm M-L, Tamminga SJ, Alleaume C, et al. Work-related outcomes in self-employed cancer survivors: A European multi-country study. Journal of Occupational Rehabilitation. 2019;29(2):361-74.

402. Trevino RA, Vallejo L, Hughes DC, Gonzalez V, Tirado-Gomez M, Basen-Engquist K. Mexican-American and Puerto Rican breast cancer survivors' perspectives on exercise: Similarities and differences. Journal of Immigrant and Minority Health. 2012;14(6):1082-9.

403. Tsai W, Wu IH, Lu Q. Acculturation and quality of life among Chinese American breast cancer survivors: The mediating role of self-stigma, ambivalence over emotion expression, and intrusive thoughts. Psycho-Oncology. 2019;28(5):1063-70.

404. Tsai W, Wu IHC, Lu Q. Acculturation and quality of life among Chinese American breast cancer survivors: The mediating role of self-stigma, ambivalence over emotion expression, and intrusive thoughts. Psycho-Oncology. 2019;28(5):1063-70.

405. Tsai W, Zavala D, Gomez S. Using the Facebook Advertisement Platform to Recruit Chinese, Korean, and Latinx Cancer Survivors for Psychosocial Research: Web-Based Survey Study. Journal of medical Internet research. 2019;21(1):e11571.

406. Tsai W, Zhang L, Park JS, Tan YL, Kwon SC. The importance of community and culture for the recruitment, engagement, and retention of Chinese American immigrants in health interventions. Translational Behavioral Medicine. 2021;11(9):1682-90.

407. Tsukinoki R, Murakami Y. Non-communicable disease epidemic: epidemiology in action (EuroEpi 2013 and NordicEpi 2013): Aarhus, Denmark from 11 August to 14 August 2013. European Journal of Epidemiology. 2013;28(1):1-270.

408. Velazquez A, Trejo E, Zheng J, Quezada-Perez G, Levine K, Friedlander T, et al. Care-ing for spanish-speaking cancer patients: the role of a latino cancer support group in a safety-net hospital. Supportive Care in Cancer. 2021;29(SUPPL 1):S222-S3.

409. Victorson D, Yao R, Murphy K, Parthasarathy S, Horowitz B, Sauer C, et al. Improvement in depressive symptoms following a 12-week yoga intervention predicts decreased inflammation at 24 weeks: Findings from a nonrandomized trial with obese, monolingual Spanish-Speaking breast cancer survivors. Global Advances in Health and Medicine. 2020;9:6-7.

410. Wang C, Lu Q. Benefit finding and depressive symptoms: The role of socioeconomic status and positive affectamong immigrant cancer survivors. Cancer Epidemiology Biomarkers and Prevention Conference: 11th AACR Conference on the Science of Cancer Health Disparities in Racial/Ethnic Minorities and the Medically Underserved New Orleans, LA United States. 2020;29(6 SUPPL 1).

411. Wang C, Lu Q. Socioeconomic status and quality of lifeamong Chinese American breast cancer survivors: Therole of post-traumatic growth. Cancer Epidemiology Biomarkers and Prevention Conference: 12th AACR Conference on the Science of Cancer Health Disparities in Racial/Ethnic Minorities and the Medically Underserved San Francisco, CA United States. 2020;29(6 SUPPL 2).

412. Wang J, Gomez SL, Brown R, Allen L, Huang E, Mandelblatt J. Ethnic variations in physical symptoms explained by different follow-up care communication and socioeconomic well-being between Chinese and Caucasian breast cancer survivors. Psycho-Oncology. 2015;2):26.

413. Wang JH, Adams IF, Pasick RJ, Gomez SL, Allen L, Ma GX, et al. Perceptions, expectations, and attitudes about communication with physicians among Chinese American and non-Hispanic white women with early stage breast cancer. Supportive Care in Cancer. 2013;21(12):3315-25.

414. Wang JH-y, Adams IF, Tucker-Seeley R, Gomez SL, Allen L, Huang E, et al. A mixed method exploration of survivorship among Chinese American and non-Hispanic White breast cancer survivors: The role of socioeconomic well-being. Quality of Life Research: An International Journal of Quality of Life Aspects of Treatment, Care & Rehabilitation. 2013;22(10):2709-20.

415. Wang JH-y, Gomez SL, Brown RL, Davis K, Allen L, Huang E, et al. Factors associated with Chinese American and White cancer survivors' physical and psychological functioning. Health Psychology. 2019;38(5):455-65.

416. Wang JHy, Adams I, Huang E, Ashing-Giwa K, Gomez SL, Allen L. Physical distress and cancer care experiences among Chinese-American and non-Hispanic White breast cancer survivors. Gynecologic Oncology.

417. Wang JHY, Adams I, Tucker-Seeley R, Gomez S, Allen L, Pasick R. A mixed method exploration of survivorship among Chinese-American and non-hispanic white patients: The role of socioeconomic well-being. Psycho-Oncology. 2013;2):38-9.

418. Wang JHY, Adams IF, Pasick RJ, Gomez SL, Allen L, Ma GX, et al. Perceptions, expectations, and attitudes about communication with physicians among Chinese American and non-Hispanic white women with early stage breast cancer. Supportive Care in Cancer. 2013;21(12):3315-25.

419. Wang JHY, Gomez SL, Brown RL, Davis K, Allen L, Huang E, et al. Factors associated with Chinese American and white cancer survivors' physical and psychological functioning. Health Psychology. 2019;38(5):455-65.

420. Wang JHY, Pasick RJ, Gomez SL, Mandelblatt JS, Brown RL, Allen L, et al. Modeling of socioeconomic wellbeing, follow-up care communication, stress appraisal, coping, and physical functioning among Chinese and non-Hispanic white breast cancer survivors. Cancer Epidemiology Biomarkers and Prevention Conference: 7th AACR Conference on the Science of Health Disparities in Racial/Ethnic Minorities and the Medically Underserved San Antonio, TX United States Conference Publication:. 2015;24(10 SUPPL. 1).

421. Warmoth K, Cheung B, You J, Yeung NC, Lu Q. Exploring the social needs and challenges of Chinese American immigrant breast cancer survivors: A qualitative study using an expressive writing approach. International Journal of Behavioral Medicine. 2017;24(6):827-35.

422. Warmoth K, Cheung B, You J, Yeung NCY, Lu Q. Exploring the Social Needs and Challenges of Chinese American Immigrant Breast Cancer Survivors: a Qualitative Study Using an Expressive Writing Approach. International journal of behavioral medicine. 2017;24(6):827-35.

423. Warmoth K, Wong CC, Chen L, Ivy S, Lu Q. The role of acculturation in the relationship between self-stigma and psychological distress among chinese american breast cancer survivors. Psychology, Health & Medicine. 2020:No Pagination Specified.

424. Warmoth K, Yeung NCY, Xie J, Feng H, Loh A, Young L, et al. Benefits of a Psychosocial Intervention on Positive Affect and Posttraumatic Growth for Chinese American Breast Cancer Survivors: A Pilot Study. Behavioral Medicine. 2020;46(1):34-42.

425. Weiner D, Burhansstipanov L, Krebs LU, Restivo T. From survivorship to thrivership: native peoples weaving a healthy life from cancer. Journal of Cancer Education. 2005;20:28-32.

426. Wen KY, Fang CY, Ma GX. Breast cancer experience and survivorship among Asian Americans: A systematic review. Journal of Cancer Survivorship. 2014;8(1):94-107.

427. Wiese D, Lynch SM, Stroup AM, Maiti A, Harris G, Vucetic S, et al. Examining socio-spatial mobility patterns among colon cancer patients after diagnosis. SSM - Population Health. 2022;17 (no pagination).

428. Wiley G, Piper A, Phyllis Butow AM, Schofield P, Douglas F, Roy J, et al. Developing Written Information for Cancer Survivors from Culturally and Linguistically Diverse Backgrounds: Lessons Learnt. Asia-Pac. 2018;5(1):121-6.

429. Wilson S, Saracco JF, Krikun R, Flockhart DTT, Godwin CM, Foster KR. Drivers of demographic decline across the annual cycle of a threatened migratory bird. Scientific reports. 2018;8(1):7316.

430. Winters AC, Viramontes M, Buch A, Najarian L, Yum J, Yang L, et al. Older Patients with Hepatocellular Carcinoma Are Less Knowledgeable about Survivorship Issues: Outcomes from a Survey-based Study. Journal of Clinical Gastroenterology. 2021;55(1):88-92.

431. Wittmann D, Northouse L, Foley S, Gilbert S, Wood DP, Jr., Balon R, et al. The psychosocial aspects of sexual recovery after prostate cancer treatment. International Journal of Impotence Research. 2009;21(2):99-106.

432. Wong I, Yoo G, Levine E, Aviv C, Ewing C, Au A. Supportive networks and social support among Chinese immigrant breast cancer survivors. Psycho-Oncology. 2009;1):S87-S8.

433. Wong-Kim E, Sun A, DeMattos MC. Assessing cancer beliefs in a Chinese immigrant community. Cancer control : journal of the Moffitt Cancer Center. 2003;10(5 Suppl):22-8.

434. Wong-Kim E, Sun A, Merighi JR, Chow EA. Understanding quality-of-life issues in Chinese women with breast cancer: a qualitative investigation. Cancer control : journal of the Moffitt Cancer Center. 2005;12 Suppl 2:6-12.

435. Wu CS, Warmoth KM, Cheung B, Loh A, Young L, Lu Q. Successful Strategies for Engaging Chinese Breast Cancer Survivors in a Randomized Controlled Trial. Translational Issues in Psychological Science. 2019;5(1):51-61.

436. Yamada R. Spirituality and psychological well-being among Asian American breast cancer survivors. Dissertation Abstracts International: Section B: The Sciences and Engineering. 2011;71(9-B):5810.

437. Yan AF, Stevens P, Holt C, Walker A, Ng A, McManus P, et al. Culture, identity, strength and spirituality: A qualitative study to understand experiences of African American women breast cancer survivors and recommendations for intervention development. European Journal of Cancer Care. 2019;28(3):N.PAG-N.PAG.

438. Yanez B, Maggard Gibbons M, Moreno PI, Jorge A, Stanton AL. Predictors of psychological outcomes in a longitudinal study of Latina breast cancer survivors. Psychology and Health. 2016;31(11):1359-74.

439. Yanez B, Oswald LB, Baik SH, Buitrago D, Iacobelli F, Perez-Tamayo A, et al. Brief culturally informed smartphone interventions decrease breast cancer symptom burden among Latina breast cancer survivors. Psycho-Oncology. 2020;29(1):195-203.

440. Yang P. Maximizing quality of life remains an ultimate goal in the era of precision medicine: Exemplified by lung cancer. Precision Clinical Medicine. 2019;2(1):8-12.

441. Yeung N, Dai J, Zheng D, Man J, Loh A, Young L, et al. Exploration of culturally unique needs and challenges experienced by chinese breast cancer survivors living in the United States: A qualitative study using expressive writing approach. Psycho-Oncology. 2011;1):92.

442. Yi J, Luong K, Yeoung K. Information seeking behaviors among breast cancer survivors with limited English skills. Psycho-Oncology. 2010;2):S198.

443. Yi JK, Swartz MD, Reyes-Gibby CC. English proficiency, symptoms, and quality of life in Vietnamese-and Chinese-American breast cancer survivors. Journal of Pain and Symptom Management. 2011;42(1):83-92.

444. Yilmaz NG, Sungur H, van Weert JCM, van den Muijsenbergh M, Schouten BC. Enhancing patient participation of older migrant cancer patients: needs, barriers, and eHealth. Ethnicity & Health. 2020:1-24.

445. Yoo G, Fung LC, Levine E, Tang C. Developing a peer support program for Chinese immigrant women breast cancer patients. Psycho-Oncology. 2011;1):65.

446. Young J, Durcinoska I, Jorgensen M, Solomon M. Survivorship care for people with colorectal cancer in Australia: A population-based survey. Supportive Care in Cancer. 2015;1):S354-S5.

447. Young JM, Durcinoska I, DeLoyde K, Solomon MJ. Patterns of follow up and survivorship care for people with colorectal cancer in new South Wales, Australia: A population-based survey. BMC Cancer. 2018;18(1) (no pagination).

448. Zebrowski CM, Vega M, Llorente AM. Cultural and linguistic issues in the assessment and treatment of pediatric cancer survivors. Handbook of long term care of the childhood cancer survivor. 2015:299-313.

449. Zeissig SR, Singer S, Koch L, Zeeb H, Merbach M, Bertram H, et al. Utilisation of psychosocial and informational services in immigrant and non-immigrant German cancer survivors. Psycho-Oncology. 2015;24(8):919-25.

450. Zeng YC, Ching SS, Loke AY. Quality of life measurement in women with cervical cancer: implications for Chinese cervical cancer survivors. Health and Quality of Life Outcomes. 2010;8:30.

451. Zhang D, Hu H, Shi Z, Li B. Perceived needs versus predisposing/enabling characteristics in relation to internet cancer information seeking among the US and Chinese public: Comparative survey research. Journal of Medical Internet Research Vol 23(1), 2021, ArtID e24733. 2021;23(1).
